# Coinfections and their molecular consequences in the porcine respiratory tract

**DOI:** 10.1186/s13567-020-00807-8

**Published:** 2020-06-16

**Authors:** Georges Saade, Céline Deblanc, Juliette Bougon, Corinne Marois-Créhan, Christelle Fablet, Gaël Auray, Catherine Belloc, Mily Leblanc-Maridor, Carl A. Gagnon, Jianzhong Zhu, Marcelo Gottschalk, Artur Summerfield, Gaëlle Simon, Nicolas Bertho, François Meurens

**Affiliations:** 1 INRAE, Oniris, BIOEPAR, 44300 Nantes, France; 2 Swine Virology Immunology Unit, Ploufragan-Plouzané-Niort Laboratory, ANSES, BP 53, 22440 Ploufragan, France; 3 University of Rennes 1, Rennes, France; 4 Mycoplasmology, Bacteriology and Antibiotics Resistance Unit, Ploufragan-Plouzané-Niort Laboratory, ANSES, BP 53, 22440 Ploufragan, France; 5 Epidemiology Health and Welfare Unit, Ploufragan-Plouzané-Niort Laboratory, ANSES, BP 53, 22440 Ploufragan, France; 6 Institute of Virology and Immunology (IVI), Sensemattstrasse 293, 3147 Mittelhäusern, Switzerland; 7 Swine and Poultry Infectious Diseases Research Center, Faculty of Veterinary Medicine, University of Montreal, St-Hyacinthe, QC Canada; 8 College of Veterinary Medicine, Comparative Medicine Research Institute, Yangzhou University, Yangzhou, 225009 China; 9 Joint International Research Laboratory of Agriculture and Agri-Product Safety, Yangzhou, 225009 China; 10 Department of Infectious Diseases and Pathobiology, University of Bern, 3012 Bern, Switzerland

## Abstract

Understudied, coinfections are more frequent in pig farms than single infections. In pigs, the term “Porcine Respiratory Disease Complex” (PRDC) is often used to describe coinfections involving viruses such as swine Influenza A Virus (swIAV), Porcine Reproductive and Respiratory Syndrome Virus (PRRSV), and Porcine CircoVirus type 2 (PCV2) as well as bacteria like *Actinobacillus pleuropneumoniae*, *Mycoplasma hyopneumoniae* and *Bordetella bronchiseptica*. The clinical outcome of the various coinfection or superinfection situations is usually assessed in the studies while in most of cases there is no clear elucidation of the fine mechanisms shaping the complex interactions occurring between microorganisms. In this comprehensive review, we aimed at identifying the studies dealing with coinfections or superinfections in the pig respiratory tract and at presenting the interactions between pathogens and, when possible, the mechanisms controlling them. Coinfections and superinfections involving viruses and bacteria were considered while research articles including protozoan and fungi were excluded. We discuss the main limitations complicating the interpretation of coinfection/superinfection studies, and the high potential perspectives in this fascinating research field, which is expecting to gain more and more interest in the next years for the obvious benefit of animal health.

## Introduction

Bacterial and viral respiratory diseases are a major health issue in species reared under confined conditions in large groups. Most often multiple infectious agents are involved in the development of these clinical conditions making unsuited the common reductionist approach of host–pathogen interactions by the study of single infection [1]. Infection by more than one type of pathogen (viruses, bacteria and parasites amongst others) is described as a mixed infection. However, the term coinfection is frequently used to describe concomitant infection of a cell or a host by separate pathogens [2]. Since in the literature the definitions of coinfection and mixed infection have been both used to describe the same events, we will use the term “coinfection” in the current review. Additionally, in virology, the term superinfection is used if one virus infects the cell or the host before infection by the second superinfecting virus. We will also use the term “superinfection” in the review. Finally, an opportunistic pathogen is usually considered as a pathogen that would not have infected animals in absence of the primary infection, or alternatively, “pathogen” that would have been asymptomatic in the absence of the primary infection. In some studies, however, the use of the terms “coinfection” is not suitable and “superinfection” should be used instead, as we will see later. This semantic point is responsible for a lot of confusion and makes comparisons between studies sometimes tricky.

The outcome of any coinfection or superinfection can be affected by the interactions taking place between the infectious agents, the nature of the cell/host, adverse environmental and management conditions, intestinal and respiratory microbiomes, and the triggered immune response—innate and adaptive—developed afterwards [2, 3]. When occurring at the same time or with a delay, infections can impact the virulence of causative pathogens with subsequent consequences on the host immune response and its ability to clear the infections [2]. The first contact with a pathogen can change the cell/host response against any other second pathogen, possibly causing a more virulent infection, reducing its severity or suppressing it completely [4]. Thus, different scenarios concerning the pathogen interactions can be observed, the first infectious agent can promote the second one, attenuate its effects or simply prevent its establishment. Conversely, the second pathogen may also influence the first one directly or indirectly.

Coinfections have been described in both humans and animals [1, 2]. Moreover, bacterial and viral infections might be followed by secondary bacterial or viral infections, which in some cases are responsible for the pathology development and the observed clinical signs. In this review, the current knowledge regarding frequent coinfections that occur in the porcine respiratory tract and particularly in the lungs are reviewed. When possible, we focused on the interactions between the mentioned pathogens and the various mechanisms justifying these interactions and their consequences on the host’s response. We especially discussed coinfections involving main bacteria and viruses associated with the so-called porcine respiratory diseases, excluding coinfections involving parasites and fungi (including their metabolites, such as mycotoxins). Moreover, we do not discuss the impact of adverse environmental and management conditions which have been shown to be of major importance in the modulation of respiratory infections’ severity [3].

## Porcine respiratory disease complex and the associated pathogens

Respiratory diseases have been formally described in pigs as early as the 1960′s [5] and several studies have been carried out to identify associated agents. The role of the infectious pathogens has been assessed by using two main approaches: direct research of the pathogens (by culture or Polymerase Chain Reaction—PCR for instance) from tissue samples of diseased (acute or chronic stage) and non-diseased pigs or indirect detection by serological tests to look for antibodies produced after exposure to specific pathogens. These studies indicated that frequently under field conditions, several infectious pathogens are simultaneously detected from lung lesions (see [6–8] amongst others). Combinations of several infectious pathogens in particular bacteria and viruses frequently occur and are responsible for respiratory diseases in pigs reared under confined conditions in large communities [1]. However, the type of combinations and associated infectious agents change over time with the emergence of new viral pathogens generally complicating disease severity [e.g. new strains of Porcine Reproductive and Respiratory Syndrome Virus (PRRSV), new types of Porcine CircoVirus (PCV), new strains of Porcine Respiratory alphaCoronaVirus (PRCoV) and new reassortants of swine Influenza A Virus (swIAV)].

Causative respiratory infectious agents can be divided into primary and secondary or opportunistic pathogens. Primary pathogen being defined here as pathogen that can infect the animal as first unique pathogen and then facilitate secondary or opportunistic coinfection. These primary pathogens include common bacteria such as highly virulent *Actinobacillus* (*A*.) *pleuropneumoniae*, *M. hyopneumoniae*, *Bordetella* (*B.*) *bronchiseptica* in young piglets and common viruses such as swIAV [1]. PRRSV and PCV2 are not strictly respiratory pathogens as swIAV, however, since they also frequently affect the respiratory system and since they can act as facilitators of secondary respiratory infections, they must be considered too. Other primary pathogens such as Aujeszky’s Disease Virus (ADV) and PRCoV are reported but they are far less frequently encountered today or they have less impact on porcine health [1]. Then, some viruses like the porcine cytomegalovirus can also inhibit host immune functions—particularly the action of T lymphocytes—and promote respiratory diseases such as the porcine reproductive and respiratory syndrome [9]. Among the secondary pathogens common bacteria such as lower virulence strains of *A. pleuropneumoniae*, *A. suis*, *Glaesserella parasuis*, *Pasteurella multocida*, and *Streptococcus* (*S.*) *suis* are reported. Together primary and secondary pathogens are involved in the “Porcine Respiratory Disease Complex” (PRDC) [10].

Several studies have assessed the nature of the infectious agents directly or indirectly associated with respiratory diseases in pigs [7, 8, 11, 12]. In one of these studies involving breeding sows in five French farrow-to-finish herds [12], results indicated that *S. suis*, a secondary pathogen, was quite widespread among sows—67.1% of the animals being positive using a PCR assay—and PCV2 and swIAV infections were highly prevalent (75% of the sows with antibodies against PCV2 and between 91.7% and 100% of the sows with antibodies against swIAV). Other infectious agents such as *A. pleuropneumoniae*, *G. parasuis* and *P. multocida* were detected in 31%, 25%, and 23% of the sows, respectively [12]. In another study evaluating infectious agents associated with respiratory diseases in 125 farrow-to-finish pig herds in France, it has been shown that *M. hyopneumoniae*, PRRSV, and swIAV subtype H1N1 were the major pathogens involved in pneumonia-like gross lesions [8]. For extensive pleuritis, PRRSV was frequently associated with *A. pleuropneumoniae* [8, 12]. Regarding bacteria associated with lung lesions in 3731 French slaughter pigs [8], a report mentioned lesions of pneumonia and pleuritis as the most frequent lesions. In these lesions, bacteria such as *M. hyopneumoniae*, *P. multocida*, *A. pleuropneumoniae*, *S. suis*, and *G. parasuis* were detected in 69.3%, 36.9%, 20.7%, 6.4%, and 0.99% of the lungs, respectively [13]. In a retrospective analysis of the etiologic agents associated with respiratory diseases in pigs in USA, two or more infectious agents were identified in 88.2% of the analyzed cases [7]. PRRSV (35.4% of the samples), *P. multocida* (31.6%), *M. hyopneumoniae* (27%), swIAV (22.2%), *G. parasuis* (22.0%) and PCV2 (18.6%) were the infectious agents most frequently encountered [7]. In Korean pigs, PRRSV and PCV2 were frequently identified associated or not to various bacteria such as *S. suis* (25.2%), *M. hyopneumoniae* (20.1%), *P. multocida* (12.9%), and *A. pleuropneumoniae* (5%) [11].

Below we review the main primary pathogens as defined above, common viruses such as PRRSV, PCV2, swIAV, PRCoV and ADV as well as bacteria like *A. pleuropneumoniae*, *M. hyopneumoniae* and *B. bronchiseptica*. Conversely, other pathogens involved in the PRDC are not presented in the following sections while considered in Additional file 1 presenting the different coinfections’ situations.

### Porcine reproductive and respiratory syndrome virus

PRRSV is an enveloped single stranded positive RNA virus belonging to the *Arteriviridae* family. Two different species, PRRSV-1 (also known as Betaarterivirus suid 1), from European origin, and PRRSV-2 from American origin, are now distinguished [14]. This enveloped virus replicates mainly or exclusively in macrophages such as Alveolar Macrophages (AMs), but also macrophages from the nasal mucosa and Pulmonary Intravascular Macrophages (PIMs) [15, 16]. In vitro, PRRSV can also replicate in cultured monocytes and monocyte-derived cells including macrophages [17] and in vitro-derived Dendritic Cells (DCs) generated either from Bone Marrow hematopoietic cells (BMDCs) or blood Monocytes (MoDCs), depending on the in vitro culture conditions [18, 19]. However, such in vitro generated DCs are not representative of in vivo primary DCs which do not seem to be permissive to viral replication [20]. In fact, MoDC and BMDC (at least when generated using Granulocyte Macrophage Colony-Stimulating Factor, GM-CSF) although possessing functional overlaps with the DC family, do not represent *bona fide* DCs, which represent an own lineage of hematopoietic cells distinct from the monocytic lineage [21]. Different cell surface molecules are involved in PRRSV entry and infection of cells: heparan sulfate, porcine sialoadhesin—also known as sialic acid-binding immunoglobulin-type lectin 1 (Siglec-1), Siglec-10, CD151 and CD163 [22, 23]. Heparan sulfate is a GlycosAminoGlycan (GAG) that seems to play a modest or secondary role in PRRSV infection since the blocking of this receptor on AMs induced only a mild decrease in PRRSV infectivity. Moreover, this effect was not observed with all the PRRSV isolates tested, suggesting that the involvement of heparan sulfate depends on the antigenic diversity of PRRSV [22]. Siglec-1/CD169 is a member of the sialic acid-binding lectins (Siglecs) family and is expressed on macrophages [22] and Siglec-10 has been identified as an alternative receptor to Siglec-1 [23]. Binding of PRRSV to Siglecs induces its internalisation by clathrin-mediated endocytosis. Expression of recombinant porcine sialoadhesin is sufficient to induce the internalisation of PRRSV by non-permissive cells, but not replication [24]. CD163 is a scavenger receptor involved in PRRSV infection [22]. Its expression on non-permissive cells makes them susceptible to infection with PRRSV and allows productive replication of the virus [22]. Moreover, CD169-KO animals are still susceptible to PRRSV-2 infection [22], whereas CD163-KO animals are resistant to PRRSV-1 and PRRSV-2 [25, 26]. Finally, MYH9 has been recently identified as an indispensable partner of CD163 for PRRSV cell entry for both PRRSV-1 and PRRSV-2 [27].

PRRS clinical signs can be nearly absent to severe depending on the considered PRRSV species and strains. When observed, there are, amongst the most frequent, lethargy, dyspnea, tachypnea, as well as a reproductive disease [16]. PRRSV can persist in infected pigs for several months after the initial infection particularly in lymphoid tissues and has the ability to alter the host’s immune system to escape it (for review see [16]). PRRSV interferes with the porcine innate immune response through downregulation of type I InterFeroNs (IFNs—IFNα and IFNβ mostly), which are cytokines known for their antiviral properties [28]. PRRSV-infected macrophages also had a reduced capacity to produce the pro-inflammatory cytokines TNFα and IL1β [28] and the production of the anti-inflammatory cytokine IL10 was found enhanced during infection [29]. Nevertheless, the role of cytokine modulation during PRRS is unclear considering that the effects appeared to depend on the PRRSV species, as well as on the PRRSV isolates, since opposite results can be found with different PRRSV strains [30, 31]. In fact, some PRRSV-2 isolates were shown to enhance IFNα production while other PRRSV-1 isolates suppressed it. Results seemed also very variable for the immunoregulatory IL10 along different isolates of PRRSV-1 [30, 31], making general conclusions about how PRRSV alters innate immune responses difficult. PRRSV impact on adaptive cellular immunity seems also to be highly variable according to the species and the strain [20]. Conversely, whereas non-protective antibody response against the viral nucleocapsid is found within a week post-infection, neutralizing antibodies appearance is highly delayed for all PRRSV species and strains, appearing only after 3 or 4 weeks of infection and peaking even later [16].

### Porcine circovirus type 2

PCV2 is a naked circular single stranded DNA virus belonging to the *Circoviridae* family and responsible for Porcine CircoVirus Disease (PCVD). The attachment of PCV2 to target cells occurs through chondroitin sulfate B and probably other receptors [32]. Internalisation is not fully known but it does not seem to involve a specific receptor and the GAGs could mediate internalisation and binding to the target cells [33]. Most of the time the infection is subclinical but in some circumstances such as coinfections with other respiratory pathogens it can cause the Post-weaning Multisystemic Wasting Syndrome (PMWS), clinically characterized by wasting respiratory disease, and enteritis [34]. Infection with PCV2 can occur in utero, resulting in stillborn piglets and mummified fetuses, or death at different ages after birth [34]. In young and older animals, PCV2 was found in cells expressing monocytes (CD14^+^), and T and B cells (CD4^+^, CD8^+^, IgM^+^) markers [35]. Further results showed that active replication of the virus was supported by T and B cells, with enhanced replication in proliferative cells [36]. In vitro, PCV2 can also infect many other cell types including endothelial cells, gut epithelial cells, fibrocytes, and DCs [37]. In DCs the virus seems to persist and remain infective for a prolonged period without replication indicating that these cells might serve as a vehicle for virus spread in the host [38]. PMWS is characterized by the depletion of lymphoid cells affecting T cells, B cells, and NK cells [39]. This lymphopenia was also associated with impaired responses of Peripheral Blood Mononuclear Cells (PBMCs) to mitogen stimulation with lower levels of IL2, IFNγ, and IL4 production compared to PBMCs from non-infected pigs [40]. Another feature of PMWS is an elevated level of IL10 found in lymphoid organs, especially in the T cells rich areas [41]. IL10-mediated immunosuppression could play an important role in the PCV2 infection and the development of PMWS. PCV2 has also the ability to alter the innate immune response [42]. Even though the virus does not productively infect DCs, evidence shows that it can interfere with the normal plasmacytoid DCs (pDCs) response. Upon stimulation with CpG-ODN, pDCs’ ability to produce IFNα and TNFα was impaired in cells previously infected with PCV2 [43]. PCV2 DNA isolated from infected cells induced the suppression of pDC IFNα production [43].

### Swine influenza A virus

Influenza A viruses are enveloped single stranded negative RNA viruses belonging to the *Orthomyxoviridae* family. These enveloped viruses can infect a broad range of hosts, with pigs being one of their natural hosts (for a review see [44]). The three main IAV subtypes encountered in pigs are H1N1, H1N2, and H3N2 [44], but many genetic lineages and antigenic variants within these subtypes are co-circulating in the pig population worldwide. Subclinical infections with swIAVs are common in pigs, but they can also induce a disease similar to what is observed in humans, with upper respiratory tract distress associated with fever, cough, rhinitis, high morbidity, and low mortality [44]. The main targets of swIAVs are epithelial cells of the respiratory tract but IAVs can also non-productively infect alveolar macrophages [45]. Two major glycoproteins are present at the surface of the virus: HemAgglutinin (HA) and NeurAminidase (NA). Binding of HA with the sialic acid molecules at the surface of the host cells will induce the endocytosis of the viral particle [44]. The NA molecule plays the main role in the budding of the virus by removing the sialic acid, allowing the release of neoformed virus particles from the infected cell [44]. The innate response against the virus includes production of high levels of pro-inflammatory cytokines such as IFNα, TNFα, and IL6. DCs, in particular pDCs play an important role in this response [46]. An important observation was that the production of these cytokines correlated to the viral loads and the severity of the disease. Infection with swIAV induces cellular and humoral specific immune responses in pigs recovering from the disease and the serum IgG and the mucosal IgA can protect the animal from re-infection [44]. NS1 and PA-X are the main viral proteins that alter the innate immune response, mainly by blocking the type I IFN response [47] as well as the NLRP3 inflammasome activation [48] in infected-epithelial cells and alveolar macrophages. Finally, the main mechanisms through which the swIAV escapes the adaptive host immune system are the antigenic drift and the antigenic shift concerning mainly HA and NA which are also the two major antigenic proteins expressed on the surface of the virus and against which the neutralizing humoral response is directed [44].

### Porcine respiratory alphacoronavirus

PRCoV is an enveloped single stranded positive RNA virus belonging to the *Coronaviridae* family. In pigs, four *Alphacoronavirus*, one *Betacoronavirus* and one *Deltacoronavirus* have been described [49, 50]. Thus, most of the porcine coronaviruses are from the genus *Alphacoronavirus*. The only respiratory porcine coronavirus, PRCoV, is a variant of Transmissible Gastroenteritis Virus (TGEV) where a large 5′ region deletion (nucleotides 621–681) in the Spike gene of the virus altered the tropism and the virulence. Even if pigs have been shown to be susceptible to the first SARS-CoV (serological evidence and isolation of the virus in a pig farm in the Xiqing County of Tianjin, China) [51] they have not been successfully experimentally infected, at this stage, by SARS-CoV-2 [52]. PRCoV uses aminopeptidase-N (CD13) domain IV to enter cells [53] and replicates to high titers in the lungs (1 × 10^7^–10^8^ Tissue Culture Infectious Dose 50—TCID_50_) specifically in type 1 and 2 pneumocytes. Moreover, it can infect epithelial cells of the nares, trachea, bronchi, bronchioles, alveoli, and, occasionally, alveolar macrophages [49]. Infections with the PRCoV are usually subclinical, but there is variation between strains and some can induce a more severe disease. PrCoV can infect pigs of all ages by direct contact transmission or aerosol [49]. The clinical signs are associated to the respiratory system and are mild to severe—bronchointerstitial pneumonia—depending the strain and the context (environmental and management factors as well as the presence of other pathogens).

### Aujeszky’s disease virus or PseudoRabies Virus (PRV)

Suid herpesvirus 1, usually known as PRV or ADV is the responsible agent of Aujeszky’s disease in pigs. It is a double stranded enveloped DNA virus from the *Herpesviridae* family and *Alphaherpesvirinae* subfamily targeting respiratory and/or genital mucosae for its replication [54]. ADV has a very broad host range varying from domestic animals like pigs, cattle, goats, sheep, cats and dogs to wild animals such as ferrets, foxes, hares, raccoons, and wild deer, and where it induces different diseases [54]. Infected animals usually show fever, sneezing, coughing and vomiting accompanied occasionally with typical nervous manifestations like convulsions, aggressiveness and lack of coordination. Mortality rate can reach 100% in suckling piglets while in mature pigs the infection is inapparent or mild [54].

ADV possesses eleven types of envelope glycoproteins playing major roles in the interaction with host cells and the induction of immune response [54]. Viral binding and fusion with the plasma membrane of the target cell—epithelial cells, neurons and alveolar macrophages—are controlled by a cascade of events orchestred by glycoproteins C (gC), gB, gD, gH and gL. The binding process starts with an interaction of gC with heparin sulfate proteoglycans [54, 55]. Stabilization of this interaction is then assured by the binding of gD to specific cellular receptors known as herpesvirus entry mediators such as HveA (TNFRSF14), HveB (PRR2, nectin 2), HveC (PRR1, nectin 1), HveD (PVR, CD55), and 3-*O*-sulfated heparin sulfate [54, 56]. At this stage, Tyrosine-based or dileucine-based endocytosis in parallel with clathrin-mediated endocytosis occur by the mediation of gB, gH and gL, leading to the penetration of the capsid and the tegument into the cellular cytoplasm. Finally, the interaction of the capsid with dynein leads to the release of viral DNA into the cellular nucleus after a transport along microtubules from the periphery to the nuclear pores [55].

Porcine humoral immune response is induced by ADV and neutralizing antibodies are mainly directed against gC [57]. Specific cell mediated immune responses are also triggered and MHC class I restricted, gC-specific, cytotoxic cells are induced. ADV also alters the IFN signaling pathway by suppressing STAT1 tyrosine phosphorylation leading to an inhibition of IFN-Stimulated Genes (ISGs) expression [54, 57].

ADV may be involved in the PRDC and can be isolated alone or with other pathogens. Accordingly, a study conducted in Taiwan reported the association of ADV with PCV2 in 10.3% of the evaluated pigs using a multiplex PCR [58].

### *Actinobacillus pleuropneumoniae*

Animals affected with this Gram negative bacterium develop a pleuropneumonia characterized by fibrinohemorrhagic necrotizing bronchopneumonia and fibrinous pleuritis which can reach a high mortality rate [59, 60]. Although the disease is best known in its acute/peracute forms, subacute and/or chronic presentations with low or no mortality are highly prevalent, especially in the presence of antibiotic treatments. Many herds are subclinically infected without previous or present episodes of clinical disease and in the absence of suggestive lesions at the slaughter house. Animals are, nevertheless, carriers of the pathogen. This happens in several conventional herds which may be simultaneously infected not only with several low/intermediate virulent strains, but also, in some cases, with strains highly likely to cause disease. In the latter case, outbreaks may suddenly appear in the presence of concomitant diseases or as a consequence of changes in management and/or environment [59, 60]. Eighteen serotypes of the bacterium have been described, which can all induce disease, although clear differences in virulence have been described [59, 60]. These bacteria can be found mainly in tonsils of carrier animals; virulent strains have a tropism for the lower respiratory tract where they preferentially bind to ciliated cells of the terminal bronchioli and pneumocytes [59, 60]. Different virulence factors expressed by *A. pleuropneumoniae* are involved in the colonization and the development of the disease. Adhesion to cells could be mediated by type IV fimbriae that are expressed upon contact with respiratory epithelial cells in vitro and during lung infection [59, 60]. Adhesion of *A. pleuropneumoniae* to respiratory epithelial cells also involves the binding of bacterial lipopolysaccharides to glycosphingolipids on the surface of the cells [59, 60]. The formation of biofilm by the bacteria is likely to play an important role in the colonization of the host [61]. After attachment to the target cells, the bacteria can produce four different pore-forming exotoxins (Apx I, II, III and IV) inducing the lysis of alveolar epithelial cells, thus allowing the acquisition of nutrients by the bacteria, but also participating in the development of the lesions [60, 62]. Some of the virulence factors expressed by *A. pleuropneumoniae* interfere with the host’s immune response. The toxins Apx I, II and III induce the lysis of not only respiratory epithelial cells, but also of cells involved in the innate immune response such as macrophages and neutrophils [60, 63]. At lower concentrations, these toxins lose their lytic properties but can still impair macrophages chemotactic activity and their phagocytic abilities [64]. The capsular polysaccharides of *A. pleuropneumoniae* interfere with macrophage phagocytosis and enable resistance to complement-mediated killing [60]. *A. pleuropneumoniae* may also interfere with the antibody response by producing proteases that can degrade porcine IgA and IgG [59, 60].

### *Mycoplasma hyopneumoniae*

This cell wall-free bacterium is considered to play a primary role in PRDC and is the causative agent of porcine Enzootic Pneumonia (EP), a disease with high morbidity but low mortality rates [65]. The main pathological mechanisms involved in *M. hyopneumoniae* infections are: (i) adhesion to the ciliated cells of the tracheal epithelium inducing ciliostasis, loss of cilia and exfoliation, dysregulation of cellular homeostasis (with increased intracellular calcium concentration) and secretion of cytotoxic factors, (ii) alteration of the mucociliary tract, (iii) inflammatory reactions sometimes exacerbated and prolonged, and (iv) manipulation of the innate and adaptive immune responses [65, 66]. Among the adhesins described in *M. hyopneumoniae*, P97 is reported to be a major determinant of cell adhesion [65–67]. Several other adhesins were reported: P102 linked to P97, LppS, LppT, MgPa, P65, P76, P110, P146, P159, and P216 [65, 66]. Most adhesins are transcribed and translated during *M. hyopneumoniae* infection and then undergo post-translational cleavage to result in diverse products on the membrane surface [65, 67, 68]. The diversity of surface proteins can also derive from the variation in the number of repeats in genes encoding adhesins [69]. These mechanisms of antigenic variation enable the bacterium to escape from immune system recognition and to invade the host [66]. Adhesins can also recruit extracellular matrix components (plasminogen, fibronectin and actin amongst others), and therefore can promote invasion and inflammatory response [65, 70].

The immune response induced against *M. hyopneumoniae*, may have a double action: over-activation of the local immune response resulting in a pathologic inflammatory reaction or local immunosuppression explaining the chronic nature of the associated pathologies [65, 66]. Acute *M. hyopneumoniae* infection leads to the recruitment and activation of various innate immune cells, essentially through the involvement of a large range of cytokines: IL1, IL6 and TNFα in lungs; CXCL8, IL1, IL2, IL4, IL6, TNFα and IL10 in Bronchus-Associated Lymphoid Tissue (BALT) or TracheoBronchial Lavage Fluid (TBLF) [65, 71, 72]. Some of these inflammatory cytokines (TNFα, CXCL8, IL1β, IL6) are produced chronically in the lungs and play powerful roles in apoptosis (TNFα), differentiation and chemotaxis of neutrophils (respectively, IL6 and IL8), and macrophage activation (TNFα, IL1β). Chronic infections are typically associated with intense lymphoid hyperplasia [71] and are characterized by an accumulation of IgG- and IgA-expressing plasma cells, CD4^+^ T cells, macrophages and DCs in the BALT of inflamed lung tissue [73]. Involvement of T cell activation in chronic inflammation is also supported by the presence of T-cell cytokines such as IL-2 and IL-4 in bronchoalveolar exudates [72].

In vitro studies conducted with macrophages co-cultured with *M. hyopneumoniae* highlighted a strong activation of inflammatory pathways inducing the production of cytokines and chemokines, and expression of receptors or pathways inducing cell apoptosis [65, 66, 74, 75]. Moreover, *M. hyopneumoniae* is described as an inhibitor of macrophages phagocytic activity, which may explain the chronicity of *M. hyopneumoniae* infections and the greater host susceptibility to other pathogens [65, 66, 74].

*Mycoplasma hyopneumoniae* was found to activate co-stimulatory molecule expression on *bona fide* DCs with poor TNFα production, contrasting with monocytes. Interestingly, a strong mitogenic activity for B cells was observed [76]. Altogether, these data indicate that *M. hyopneumoniae* is well sensed by the innate immune system, but the presence of immune evasion mechanisms targeting antigen presenting cells remains a possibility that needs further investigations.

Antibody responses after infection develop slowly and do not appear to correlate with protection [65, 66]. The literature on *M. hyopneumoniae* infections coupled with information from mouse models indicates that adaptive immune responses represent a fragile balance between pathogenic and protective Th- cell responses, probably belonging to the Th1 or Th17 types [65, 66].

### *Bordetella bronchiseptica*

This aerobic Gram-negative bacterium can be found in the respiratory tract of several animal species and it presents a worldwide distribution in the porcine rearing [77]. *B. bronchiseptica* has a strong tropism for ciliated cells from the respiratory tissue and is mostly detected in the apical portion of the ciliated cells of turbinates, trachea and lungs [77, 78]. It can also be found in the cytoplasm of neutrophils and macrophages and rarely in the alveolar lumen associated with small tufts of cilia [77, 78]. Hence, infected pigs show cilia loss in the bronchial and bronchiolar epithelium associated with multifocal erosion, fibrosis, and hyperplasia. Neutrophil infiltrates are noted in the peri-conchal meatus and the submucosa of the bronchioles and alveoli, while lymphocyte and plasma cell infiltrations occur at the level of the *lamina propria* [77, 78].

Cell adhesion of *B. bronchiseptica* is a multifactorial process involving two main virulence factors; Filamentous HemAgglutinin (FHA) and PeRtactiN (PRN) [77, 79]. The expression of both adhesins is controlled by the *Bordetella* virulence genes (Bvg)AS signal transduction system. FHA is an adhesin with several binding domains including a carbohydrate- recognition domain responsible of the adhesion to macrophages and ciliated epithelial cells, a heparin-binding domain that mediates the binding to sulfated polysaccharides, and an Arg-Gly-Asp domain (RGD) regulating the InterCellular Adhesion Molecule 1 (ICAM1) by epithelial cells after interaction with the NF-κB signalling pathways [79]. This RGD domain is also present in the structure of PRN and contributes to the binding process [77, 79]. On the other hand, non-opsonic adhesion mechanisms play a role in binding to the host cells such as carbohydrate-specific mechanisms and those involving sialic acid-containing compounds [77].

Virulence of the bacteria depends on the strains; therefore, clinical signs can be different going from sneezing and transient nasal discharge for moderate and non-toxic strains to bronchopneumonia and atrophy of the nasal turbinate bones for virulent strains, especially if they are associated to other bacteria such as *P. multocida* [77]. Thus, *B. bronchiseptica* is usually described as primary lung pathogen in young pigs where it causes necrohemorrhagic bronchopneumonia whereas in older pigs this bacterium is mostly known as an opportunistic pathogen contributing to the PRDC [77]. The immune response against *B. bronchiseptica* is mainly triggered by the different toxins expressed such as adenylate cyclase, tracheal cytotoxin and DermoNecrotic Toxin (DNT).

## Coinfections and superinfections and the resultant consequences for the porcine host

### Selection and exclusion criteria for considered studies

**Figure 1:**
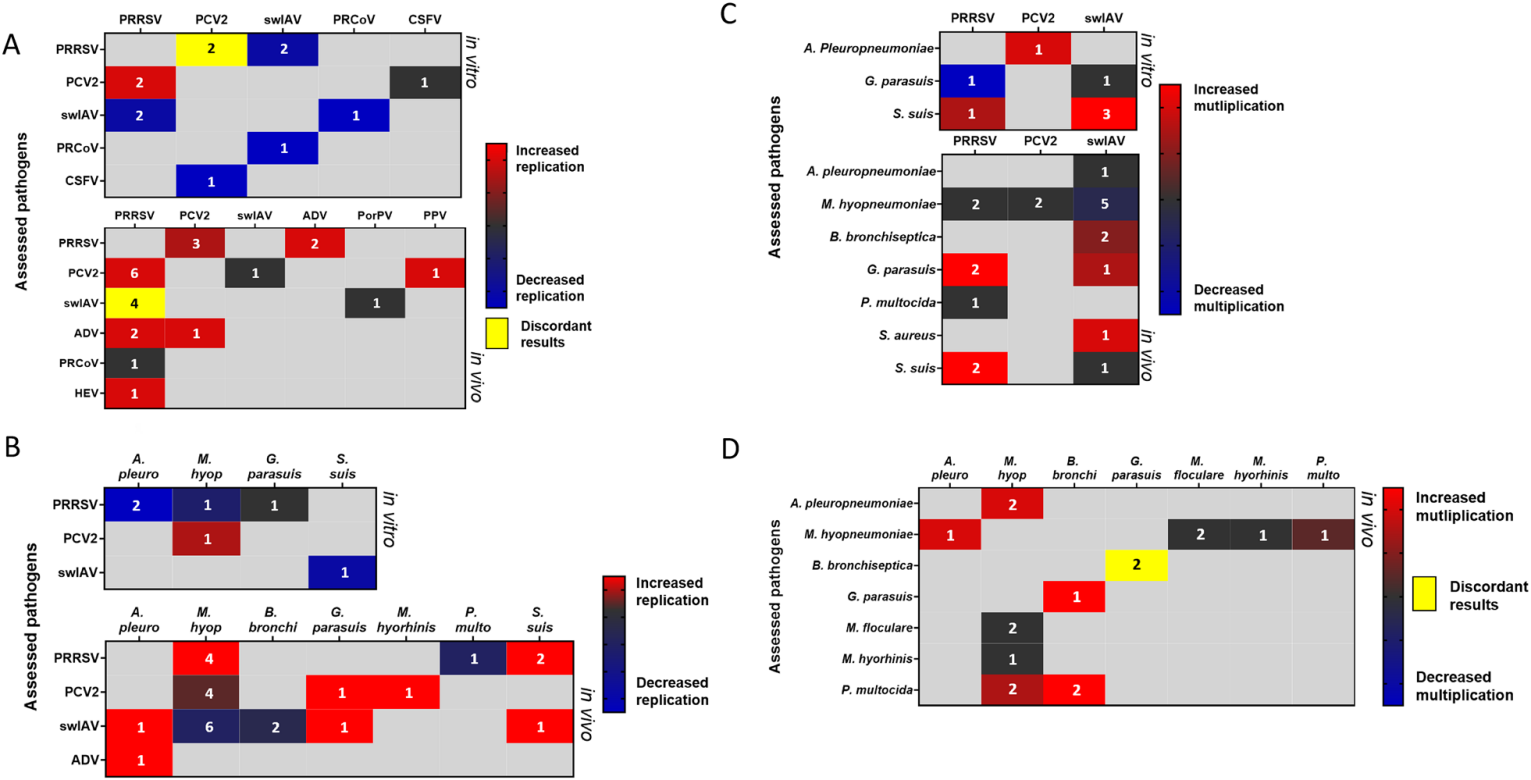
**Impact of coinfection on multiplication/replication of the microorganisms.** Heat maps depicting: **A** the impact of a secondary viral infection (top) on the replication of the virus (side) responsible of the primary infection in virus-virus dual infections (coinfections and superinfections) in vitro (up) and in vivo (down). **B** the impact of a bacterial infection on the replication of the infecting virus in virus-bacterium dual infections (coinfections and superinfections) in vitro (up) and in vivo (down). **C** the impact of a viral infection on the multiplication on the infecting bacteria in virus-bacterium dual infections (coinfections and superinfections) in vitro (up) and in vivo (down). **D** the impact of a secondary bacterial infection (top) on the multiplication of the bacteria (side) responsible of the primary infection in bacterium-bacterium dual infections (coinfections and superinfections) in vivo. The numbers shown on the maps correspond to the number of the identified studies for the same pathogen combination (see Additional file 1A and B). PRRSV: Porcine Reproductive and Respiratory Syndrome Virus, swIAV: swine Influenza A Virus, PCV2: Porcine Circovirus type 2, ADV: Aujeszky’s Disease Virus, PRCoV: Porcine Respiratory alphaCoronaVirus, CSFV: Classical Swine Fever Virus, HEV: Hepatitis E Virus, PorPV: Porcine RubulaVirus, PPV: Porcine ParvoVirus.

**Figure 2:**
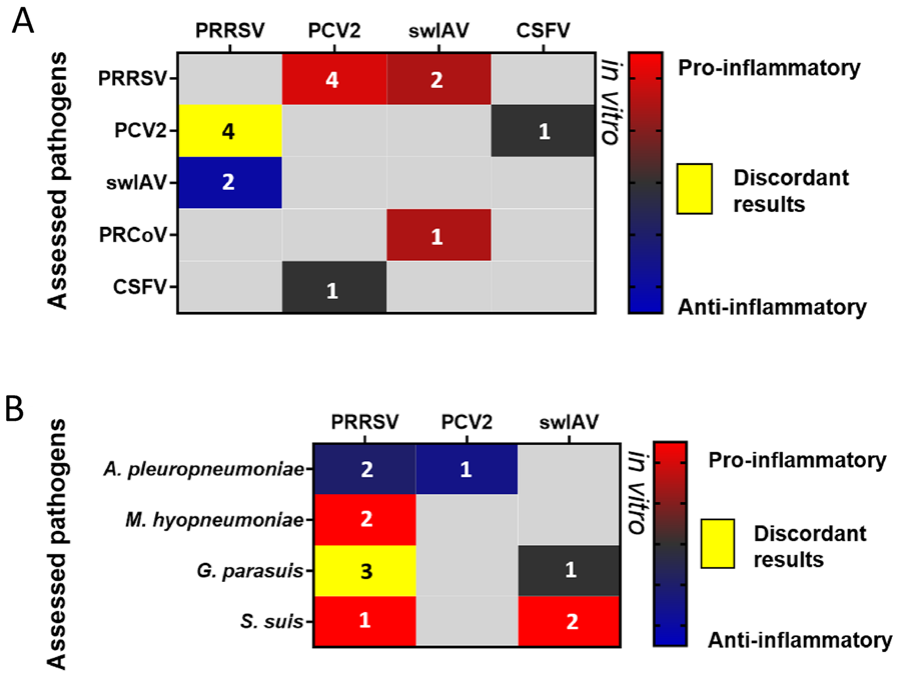
**Impact of coinfection on inflammation.** Heat maps depicting: **A** the impact of dual virus-virus infections (coinfections and superinfections) and **B** the impact of virus-bacterium infections (coinfections and superinfections) on the immune response of hosting cells (in vitro). The numbers shown on the maps correspond to the number of the identified studies for the same pathogen combination (see Additional file 1). PRRSV: Porcine Reproductive and Respiratory Syndrome Virus, swIAV: swine Influenza A Virus, PCV2: Porcine Circovirus type 2, PRCoV: Porcine Respiratory alphaCoronaVirus, CSFV: Classical Swine Fever Virus.

**Figure 3:**
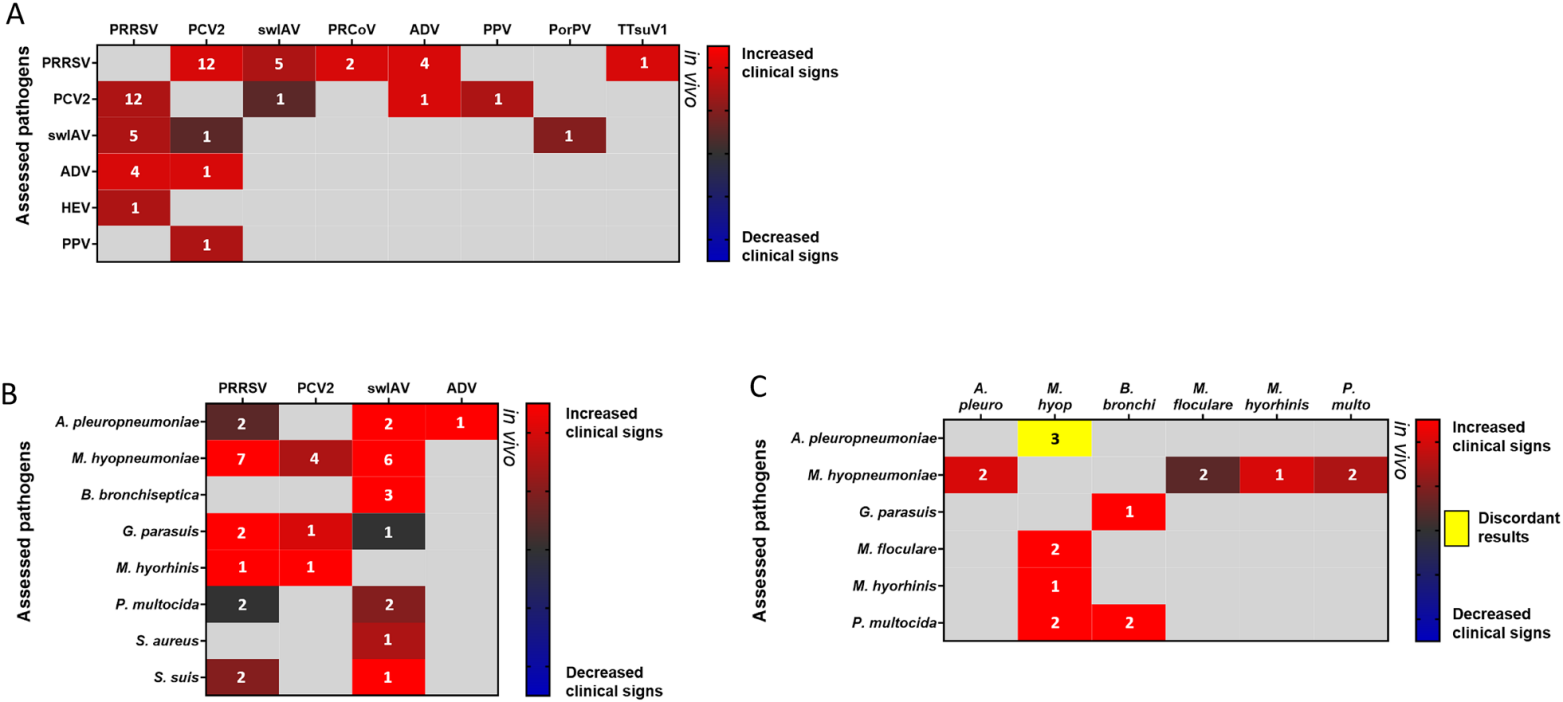
**Impact of coinfection on clinical signs.** Heat map depicting: **A** the clinical impact of dual virus-virus infections (coinfections and superinfections), **B** the clinical impact of virus-bacterium infections (coinfections and superinfections) and **C** the clinical impact of bacterium-bacterium infections (coinfections and superinfections) on the developed clinical signs (in vivo). The numbers shown on the maps correspond to the number of the identified studies for the same pathogen combination (see Additional file 1). *M. hyop*: *Mycoplasma hyopneumoniae*, *A. pleuro*: *Actinobacillus pleuropneumoniae*, *B. bronchi*: *Bordetella bronchiseptica*, *P. multo*: *Pasteurella multocida*, PRRSV: Porcine Reproductive and Respiratory Syndrome Virus, swIAV: swine Influenza A Virus, PCV2: Porcine Circovirus type 2, ADV: Aujeszky’s Disease Virus, PRCoV: Porcine Respiratory alphaCoronaVirus, HEV: Hepatitis E Virus, PorPV: Porcine RubulaVirus, PPV: Porcine ParvoVirus, TTsuV1: Torque Teno sus Virus 1.

In the following section and Additional file 1, we have used the published studies evaluating multiple infections including viral-viral, bacterial-viral, viral-bacterial and bacterial-bacterial respiratory coinfections and superinfections in swine. Both in vivo and in vitro studies comparing single to multiple infections were included. Studies evaluating vaccinations and the development of diagnostic techniques such as ELISA or qPCR were excluded as well as trials testing antiviral or antibacterial molecules when there was no clear comparison between single and multiple infections. An attempt to present a synthetic view of coinfections is depicted in the heat maps (see Figures 1, 2, 3). However, we recommend readers to refer to Additional file 1 for each coinfection couple to get a more detailed view.

In these heat maps, we were interested in the effect of the first pathogen on the multiplication of the second one (named “assessed pathogen” in the figures) and on the host immune response and/or clinical signs. These effects were evaluated and a grade from − 5 to + 5 was given to every pathogen depending on the intensity of its impact on the multiplication of the second agent and on the immune response or on the clinical signs. Negative grades were given to pathogens decreasing the multiplication of other pathogens, while positive grades were given to those inducing an increase. Similarly, negative grades were attributed to pathogens with a tendency to decrease clinical signs or immune response related to the other pathogen. Positive grades were given in case of an increase. The sum of the grades was calculated if the same pathogen combination was evaluated in several studies except in the case of discordant results. This grading was represented in the following heat maps and the number of the identified studies for the same pathogen combination is shown on the maps. In the heat maps, other pathogens, that are less associated to PRDC such as *G. parasuis*, *M. hyorhinis*, *M. floculare*, *P. multocida*, and *S. suis* or even not considered as respiratory pathogens like *Staphylococcus aureus*, Classical Swine Fever Virus, Hepatitis E Virus, Porcine RubulaVirus, PPV, and Torque Teno sus Virus 1 have also been included. Indeed, these pathogens can also impact the outcome of respiratory infections and deserve, at least, to be mentioned.

### The different types of coinfections

#### Virus–virus

Viral/viral respiratory coinfections have always had an important role in the porcine respiratory disease complex [1]. Several studies assessed the presence of two or more viral pathogens in pigs showing respiratory clinical signs in farms located in endemic regions [7, 8, 13]. The main viruses contributing to the porcine respiratory disease are swIAV, PRRSV, PCV2, and to a lower extent the PRCoV and the ADV. Due to their fast-spreading and their economic consequences, some viruses were more studied than others in the last 20 years, especially PCV2, PRRSV, and swIAV as shown in Additional file 1A and B. We will thus put more emphasis on these three viruses as causes of primary infections.

Many in vivo studies were carried out to assess the severity of the clinical signs and the development of the microscopic/macroscopic lesions. This approach enabled a comparison between coinfection/superinfection and single-infection conditions. Then, viral interference was progressively more frequently measured as a way to better understand the consequences of coinfections. In the last decades, the strong development of molecular biology and various tools enabled the evaluation of the immune response developed following polyviral infections.

In Additional file 1, the selected studies that were carried out on viral coinfections are presented from the oldest in vivo experiments to the latest in vitro and ex vivo experiments (Additional file 1A and B). This data synthesis highlights the major impact of PRRSV primary infection, which can both increase the titre of the following virus (PCV2, Hepatitis E Virus—HEV) in vitro [80] and in vivo [81–83] (Figure 1A), but can also worsen the clinical score associated to the disease (Figure 3A). Interestingly, even when the PRRSV does not increase the viral production of the other virus, as observed in coinfections involving swIAV [84] or PRCoV [85] (Figure 1A), it can also worsen the associated clinical signs. SwIAV and PCV2 as primary infectious agents have been less studied. However, it can be observed that swIAV can interfere with other virus productions (PRRSV and PRCoV) [85, 86] whereas PCV2 has some detrimental impact on the clinical outcome of secondary viral infections (PRRSV, swIAV, and PPV) [87–89] (see Additional file 1 and Figure 1A). Then, regarding the inflammation induced in coinfection conditions, various outcomes were observed depending which viruses were considered (Figure 2A).

#### Bacterium–virus and virus–bacterium

Many in vitro and in vivo experiments, with different bacterium-virus and virus-bacterium combinations, have been performed to identify the underlying mechanisms of the PRDC (see Figures 1B, C, 2B, C, and 3B). The main studies are presented in Additional file 1C and D.

Bacterium-viral coinfections can also involve various primary respiratory pathogens. Among them, the most frequently studied bacterium is *M. hyopneumoniae*, a pathogen that induces a chronic respiratory disease and can influence the outcome of a subsequent viral infection. However, mycoplasma infection needs to be already well established in the respiratory tract at the time of the viral infection to potentiate it. Indeed, *M. hyopneumoniae* inoculated to pigs simultaneously or shortly before the virus did not strongly impact the severity of the viral infections (PCV2, swIAV, PRRSV) [90–92], while its impact was clearly evidenced when inoculated 3 weeks before viral infections [93].

It is well-known that viral infections can induce an ideal environment for a bacterial superinfection through different mechanisms such as the destruction of the epithelial barrier, the over-expression of the receptors involved in the bacterial adhesion to the cells, and the alteration of the host immune response [1, 2, 94, 95]. The swIAV infection has been shown, for instance, as a way to facilitate the colonization of epithelial cells by *S. suis*, but only for the serotypes containing sialic-acid in their capsule [96]. The swIAV infection induces a loss of ciliated cells leading to the impairment of the mucociliary clearance function, but induces also the presence of the viral HA on the surface of infected cells that interacts with the sialic acid of the bacterial capsule, leading to increased adherence of *S. suis* [96, 97]. Although these swIAV effects on *S. suis* have been clearly shown in vitro, no clear in vivo impact of swIAV infection on *S. suis* pulmonary load has been described [98]. It was clearly shown that the presence of both pathogens significantly induces more inflammation than single infections [98, 99].

Overall, studies carried out in pigs showed that a bacterium-virus or a virus-bacterium coinfection frequently induces an aggravation of pulmonary lesions (Figure 3B) and a higher inflammation (Figure 2B) and immune response, with increased production of pro-inflammatory cytokines. In many bacterium-virus and virus-bacterium associations, this worsened outcome seems to be the result of additive effects from both pathogens rather than a real synergy [100, 101]. However, a potentiation of the viral infection by bacteria can also be observed in other cases, such as in the *M. hyopneumoniae*-PRRSV coinfection [102]. In that case, higher amounts of PRRSV genomes were detected in lymphoid tissue and blood [102] and a slower viral clearance was observed [75] (Figure 1B), suggesting that the recruitment of immune cells in the lung parenchyma upon established *M. hyopneumoniae* infection may provide a steady supply of susceptible cells for PRRSV [1]. Then, in porcine AMs and in the “African green monkey” (originally described as porcine origin) St-Jude Porcine Lung (SJPL) cell line, PRRSV infection has been shown to be blocked by a pre-infection with *A. pleuropneupmoniae*, this antiviral activity being due to the *A. pleuropneumoniae* metabolites [103] (Figure 1B). Given the fact that in vivo studies involving PRRSV and *A. pleuropneumoniae* did not always investigate the impact of an *A. pleuropneumoniae* pre-infection on the subsequent PRRSV infection [104, 105], as done in experiments performed in vitro [103], it cannot be easily concluded if this interference would be observed in the target species. However, in vivo, PRRSV-*A. pleuropneumoniae* interactions were reported as absent or mild [104, 105] (Figure 3B).

#### Bacterium–bacterium

In virus-bacterium coinfections, the dogma usually encountered is that viruses play an immunomodulatory role, which favors bacterial superinfections. Nevertheless, a pre-disposing effect is also described for *M. hyopneumoniae*, which promotes viral but also bacterial superinfections [65] (Figure 1D). Few studies of experimental coinfections or superinfections with *M. hyopneumoniae* and/or other bacteria involved in PRDC were performed compared to coinfections involving viruses. These studies are reported in Additional file 1E. Overall, these coinfections or superinfections induce more clinical signs and lung lesions and poorer technical performances when compared to single infections with the same infectious pathogens (Figure 3C). The bacterial-bacterial coinfections are also responsible for immune response alterations (for reviews see [106, 107]). For example, macrophages from pigs infected by *M. hyopneumoniae* decrease their phagocytosis capacity against *A. pleuropneumoniae* [60, 65]. *M. hyorhinis* and *M. flocculare*, two mycoplasmas commonly co-isolated with *M. hyopneumoniae* in gross pneumonia-like lesions, may also impact the immune response by inducing the cytotoxicity of immune cells and/or the secretion of cytokines affecting its outcome [108]. Co-stimulation of porcine BMDCs with *M. hyopneumoniae* and *M. hyorhinis* induces a strong IL12 production. In this last in vitro model, *M. hyopneumoniae* associated with *M. flocculare* reduces TNFα production compared to BMDCs stimulation by *M. flocculare* alone producing a TNFα concentration greater than that observed after stimulation with *M. hyopneumoniae* alone and *M. hyorhinis* alone [108]. Therefore, *M. flocculare* might play an initial role in pulmonary inflammation by inducing the production of TNFα by resident myeloid cells. Supplementary investigations will be needed to elucidate the role of this cytokine in the pathogenesis of the disease [108]. Other examples of bacterium-bacterium in vivo coinfections are presented in Additional file 1E.

### Mechanisms of coinfections interferences

Regarding coinfections and superinfections, most studies assessed the clinical outcome of the process but less is known about the mechanisms of interactions between pathogens and the consequences for the pathogens themselves, the infected cells and more generally for the host.

**Figure 4:**
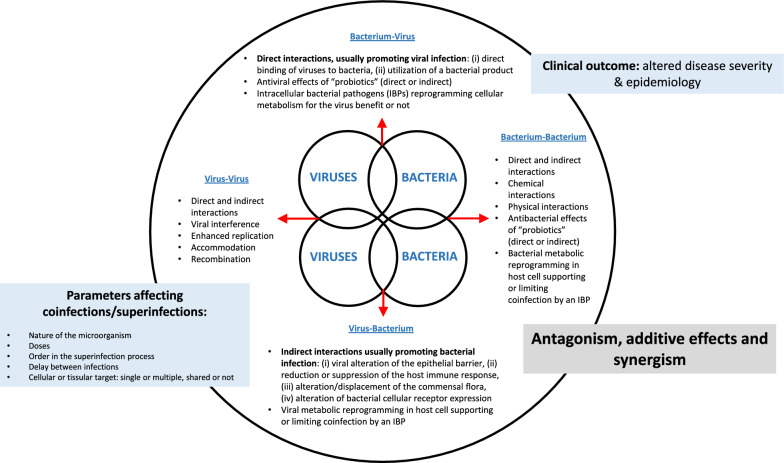
**Consequences of the different types of coinfections for the microorganisms and for the host.** In the left box some parameters enable to affect coinfections and superinfections are listed. IFN: Interferon, IBP: Intracellular Bacterial Pathogen.

The outcome of dual infection is variable depending on the antagonism, neutrality or synergy between the infectious agents. On the host side, coinfection can make the host response ineffective, and vice versa. If we look now at the possible interactions that can occur between pathogens we have to consider the nature of the infectious agents (summary provided in Figure 4). Different situations can be observed and coinfections can involve virus with virus, bacterium with virus and vice versa, and bacterium with bacterium.

#### Virus–virus interactions

Regarding virus–virus interactions, consequences are diversified and many studies looking at virus replication in coinfection situations have been carried out [2]. The first consequence of coinfection could be the so-called viral interference, a situation whereby one virus interferes with the replication of the other one making the cells resistant to the superinfecting virus [109].

The most common way for viral interference is indirect and based on the production of type 1 and 3 IFNs which induce the expression of ISGs after interacting with their cognate receptors [110, 111]. These proteins then activate numerous mediators of the cellular antiviral system that may non-specifically block the replication of viruses. They may also interfere, to a certain extent, with bacterial multiplication since IFN can also be induced by Intracellular Bacterial Pathogens (IBPs) or some extracellular bacteria [107, 112]. Nevertheless, in some situations type 1 IFNs can also increase the host susceptibility to subsequent bacterial infection [113] through impaired macrophage recruitment with a reduced CXCL1 and CXCL2 transcription [114] and a reduced IL17 [115] production. Then, there is also the non-interferon-mediated viral interference (or intrinsic interference) which is a cellular state of resistance induced by the virus to new viral infection by closely related or unrelated viruses [116]. Various mechanisms are described to explain this cellular state of direct or indirect resistance (for examples see [2]). In this type of interference, which can occur between viruses but also between viruses and bacteria [107, 117], there is a competition between pathogens for the metabolites and all the host factors that allow their multiplication. Besides the mechanisms involving a competition for common cellular factors, there are also several other mechanisms of interference described. These relies on viral Defective Interfering (DI) particles [118], RNA interference (RNAi) [119], non-specific double stranded RNA (dsRNA) [120] as well as trans-acting proteins [121]. Interference can occur at specific steps or multiple steps of the viral replication cycle (attachment, penetration, genome replication and/or budding) and can be direct or indirect. Inhibition of superinfection (superinfection exclusion and superinfection suppression) is one of several consequences that can be observed in the interference between related and unrelated microorganisms. In superinfection exclusion, an established infection interferes with a subsequent, closely related infection [2, 122]. An example of this phenomenon in pigs is the exclusion of highly virulent classical swine fever virus strain Margarita in wild boars persistently infected with this virus upon a challenge infection with the same Margarita strain [123]. Superinfection suppression is a quite close concept where this time persistently infected cells resist to a challenge with a heterologous virus [2]. Furthermore, when the host immune response—innate or adaptive—is considered in the study of the complex interactions taking place in viral coinfections, additional mechanisms of indirect interference linked to cellular and humoral cross-protection—resulting from a first viral contact with a wild-type or a vaccine strain—can be described.

Conversely, in some situations, viral coinfections can directly or indirectly result in enhanced replication and virulence for one or both pathogens as observed in several studies involving porcine viruses [80, 83, 124–126]. In other cases, coinfection/superinfection has no effects on virus replications and the viruses can coexist in a relation called accommodation [2]. Besides consequences in terms of viral replication, there are also consequences for the genetic of the viruses and their evolution through events of recombination between closely related viral genomes. Recombination, the parameters influencing it and its consequences were reviewed in RNA and DNA viruses [127, 128]. Then, as a result of all these possible interactions between viruses, the severity of the resulting disease and its epidemiology can be altered as exemplified in Additional file 1. In the pig studies, most often, however, the exact mechanisms controlling interactions between viruses were not elucidated.

#### Bacterium–virus and virus–bacterium interactions

Several mechanisms explaining bacterium–virus and virus–bacterium interactions have been identified (for reviews see [1, 94, 117]). The interactions can have either a positive or a negative impact on both pathogens depending on the bacterial and viral species involved. Usually, when the interactions are direct they promote viral infection without affecting the bacterial species [1, 94, 117]. Examples of these direct interactions are (i) direct binding of the virus to a bacterium or (ii) the utilization of a bacterial product by the virus. An example of direct interactions in the respiratory tract is the cleavage of the IAV HA into HA1 and HA2 by a *Staphylococcus aureus* protease helping the viral particle to become infectious [129]. On the contrary, when interactions are indirect they often provide an advantage to bacterial infections. Four mechanisms dealing with indirect interactions have been described: (i) viral alteration of the epithelial barrier, (ii) reduction or suppression of the host immune response, (iii) viral alteration/displacement of the microbiota, and (iv) virus-induced alteration of bacterial cellular receptor expression [94]. All these mechanisms can operate together for the benefit of the superinfecting bacteria. A typical example of these indirect interactions is provided by PCV2 and swIAV and porcine pathogenic bacteria such as *A. pleuropneumoniae* [130] and *S. suis* [96, 97, 131] where the bacteria benefit from the prior viral infections. However, bacteria can also directly benefit from a previous viral infection as observed in a study demonstrating that *Staphylococcus aureus* was able to bind viral HA [132]. The consequence of that binding was an enhanced bacterial internalisation by two mechanisms: (i) binding to HA exposed at the surface of infected cells, and (ii) binding to free extracellular virions.

In some other situations, non-pathogenic bacteria can also directly or indirectly protect the host from viral infection as typically observed with probiotic bacteria which can show antiviral activity through the binding/capture of the viruses and/or the competition for cell adhesion (for a review see [117]). This type of interaction has been frequently observed with enteric bacteria [117] and an example in pigs is the reduced infection of IPEC-J2 cells by vesicular stomatitis virus after pre-incubation of the cells with multiple probiotic bacteria [133]. An intriguing relationship is occurring between IBP and viruses where metabolic reprogramming in host cells triggered by viruses might support or conversely limit coinfection by an intracellular bacterial partner (for a review see [107]). Different possibilities can be identified in that type of interaction [107]: (i) the first pathogen can reprogram cellular metabolism related to cellular immunity and decrease the defense against the other pathogen, (ii) the metabolic changes triggered by the first pathogen can facilitate the adhesion, the penetration, and the replication of the other, and (iii) the coinfection transform the active replicative state of the first pathogen into a stable persistent state. The first possibility associated to a decrease of the cellular defense is a commonly accepted mechanism [134] while the second and the third possibilities are less experimentally demonstrated [107].

#### Bacterium–bacterium interactions

Looking at bacterium–bacterium interactions, they are extremely complex to assess because of the large diversity of the bacterial world and because little is known about the mechanisms underpinning these interactions during infections. Moreover, it is now also clear that intestinal and respiratory microbiomes affect the interactions between pathogenic bacteria and the porcine host [135]. Some examples of the complex interactions occurring in bacteria–bacteria coinfections are presented in Additional file 1E, but little is known about the mechanisms controlling these interactions. However, some mechanisms were provided above and interesting reviews dealing with that subject were published recently [106, 107] discussing the possible direct interactions between bacteria—mainly chemical and physical. Indirect interactions between bacteria were not reviewed in these articles but were discussed to some extent in other review papers focusing on polymicrobial infections [1, 136].

### Limitations of coinfection studies

The first observation coming from this review must be, even if several studies have been carried out on the subject, a lack of data about some specific coinfections and many discrepancies between studies. For instance, there are only a few in vivo studies about PCV2 in virus/virus coinfections and about PCV2 and PRRSV in virus/bacterium coinfections (Additional file 1 and heat maps in Figures 1, 2, 3). Discrepancies are not surprising because of the definition of coinfection is not always the same between studies in addition to huge variations in the coinfection parameters amongst studies.

In this review we focused on experimental (in vivo and in vitro) coinfections, it is worth to underline that these studies are inspired by field veterinarians and epidemiologists observations. However, the definition of epidemiologist coinfection can also vary between studies. Indeed, in some cases there is concomitant direct identification of two microorganisms in the same animals, sometimes in the same farms, while in other cases it is just an indirect identification of the microorganisms’ presence at some points through indirect serological assays. Moreover, as stated before, the term coinfection is sometimes used to describe some situations of superinfections where the delay can be significant.

Regarding the experimental parameters, the Multiplicities Of Infection (MOI), the strains, the potential delays between successive infections, the routes of inoculations, the types of cellular hosts considered (more or less susceptible to one of the microorganism), the genetic background (breeds) and the sanitary status (specific pathogen free or conventional breeding) of the pigs, and the readout to assess coinfection outcome varied a lot between studies. To fully compare studies, a standardization of the assays would be needed. Interestingly also, whereas in vitro studies’ usefulness is not in question, it is important to underline here that in vitro observed interplay between pathogens cannot be automatically applied in vivo. For instance, whereas *M. hyopneumoniae* decreases the PRRSV titre in vitro [75], it increases PRRSV shedding in vivo and indeed worsens the clinical signs upon coinfection [102]. Consequently, the use of intermediary settings, such as co-culture of different cell types (see [2] for examples), Precision Cut Lung Slices (PCLS) [137] or organoids [138], could help to understand the complexity of coinfections in the respiratory tissue. In vitro approaches usually consider one or a few cell types with some limitations during the evaluation process of the coinfection consequences. Some viruses can contribute to the elimination of other viruses just because of their ability to replicate faster on a particular cell type [139]. Thus, results obtained in vitro cannot mimic the field situation when both agents coinfect the same pig, providing inaccurate conclusions about the coinfection dynamics. Under such circumstances, pathogens may simply have different host cells and no longer be under direct competition for resources [140]. Besides the different interactions that infecting agents can have between them through a competition to resources, studies showed clearly that the immune system and the immunological responses can highly affect these interactions by inducing the competitive power of a pathogen or abolishing it and making it less competitive on the resource [141, 142]. The effects of the immune system (especially humoral parameters) are often not taken into consideration in selected in vitro models [140]. On the other hand, in vivo coinfection experiments have to deal with numerous constraints (health status of the animals used, cost, husbandry, and ethics amongst others) and therefore are not always easy to perform. Hence, although in vivo experiments are required in this very complex field, they surely need to be combined to in silico/in vitro/ex vivo analyses of potential interactions between pathogens. Moreover, multiple parameters of the coinfection protocols appeared difficult to set without any a priori such as the choice of the pathogen that will be inoculated first and the delay between infections. One possibility to deal with multiple parameters is to use intra-host infection mathematical modelling [143] allowing to play, at limited cost, with the different parameters of the coinfections. However, these models need to be fed with data coming from conventional in vitro experiments as well as more complex in vivo studies. The other possibility is to rely on field prevalence studies monitoring the very presence of the pathogens (isolation, PCR) instead of the sero-conversion, in order to have a clear epidemiological picture of when and where coinfections occur.

Consequently, ex vivo models such as PCLS generated from freshly sacrificed pigs [137] or organoids [138] are developing. These models are closer to mimic the in vivo situation than usual in vitro approaches, combining different types of cells and providing the pathogens with a wider range of cell hosts. However, the contribution of the immune response to the interaction between different pathogens is rarely considered [97]. Furthermore, the MOI cannot be controlled because the number of infected cells in the slice or the organoid cannot be monitored easily either.

Another limiting factor in coinfection studies is the cell regeneration, which can vary between in vivo and in vitro models. Cell regeneration can highly affect the dynamics of a coinfection, giving some pathogens extra target cells guarantying their longer existence while contributing to the clearance of others [140]. Finally, other potential technical limitations could always be discussed such as the lack of precision or sensitivity in the different diagnostic techniques especially in the presence of multiple agents. Hence, the detection of coinfecting pathogens could be compromised or reduced as compared to their detection level in the context of single infections.

## Conclusion and perspectives

As shown in this review many works have been dedicated to the study of coinfections and superinfections in pigs. Usually, when the experiments were carried out in vivo, the researchers were more interested in the clinical outcome than in the interactions occurring between pathogens. Indeed, in most of the cases the fine interactions between pathogens and especially the mechanisms behind these interactions and its potential consequences, at the molecular level, on the immune response were not studied for several reasons including technical limitations. Also, in the studies assessing the occurrence of coinfections/superinfections in the fields, coinfection identification based on molecular tools such as PCR would be more accurate than sero-prevalence approaches which are less prone to identify currently present pathogens and thus coinfective pathogens. Then, a better knowledge of each pathogen involved is crucial. We thus would like to make recommendations for future studies dealing with respiratory coinfections in pigs: (i) Authors should clearly summarize their coinfection or superinfection experimental setup—doses of pathogens, delays between infections—in their Materials and methods section; (ii) in this summary they would need to clearly present the pathogens they use and they should, as often as possible, select well-characterized strains; (iii) environmental and management conditions would need a strict control and monitoring; (iv) animal genetic and sanitary status would need to be carefully described and monitored during the study; and (v) the multiplications of all the pathogens shall be followed during the experiment using highly sensitive and specific assays. A clear description of all these parameters would help the scientific community to compare studies and progress in the understanding of the complex interactions between microorganisms.

In the last years, the concept of innate immune memory or trained immunity has gained a lot of interest. This concept is coming from old observation, in 1946 [144], recognizing that the bacterial vaccine strain “*Bacille de Calmette et Guérin*” (BCG) was protecting not only against *Mycobacterium tuberculosis* but also against antigenically different microorganism causing childhood mortality, suggesting an “adaptation” of the cellular innate immune system. Since then, many interesting studies about innate immune memory or trained immunity have been published (for a review see [145]) and it is recognized that cells such as myeloid cells, NK cells, and even epithelial cells [146] can have a higher and quicker response upon re-exposure to a pathogen. Trained immunity is accompanied by epigenetic changes and most often associated with modifications in cellular metabolism. A close look at potential epigenetic changes and cellular metabolism modifications would be of high interest in respiratory coinfection studies in the porcine species. Recently an alternative to the mechanism of trained immunity in resident lung innate immune cells named “epigenetic legacy” has been described [147]. In that study, the authors demonstrated that following IAV clearance and clinical recovery (1-month post-infection), mice were better protected from *Streptococcus pneumoniae* infection by adult bone-marrow-derived AMs displaying transient transcriptional and epigenetic distinct profiles. This newly described consequence of a first viral infection also needs additional studies about PRDC with an identification of the mechanisms shaping the complex interactions between pathogens.

## Supplementary information

**Additional file 1. Studies about coinfections in the pig respiratory tract and their consequences.**
