# Supplementary material for: Coinfections and their molecular consequences in the porcine respiratory tract
Source: Vet Res. 2020 Jun 16;51:80. doi: 10.1186/s13567-020-00807-8 (PMC7296899; doi:10.1186/s13567-020-00807-8)
Supplement: Supplementary file 1 — Additional file 1. Studies about coinfections in the pig respiratory tract and their consequences. [file 13567_2020_807_MOESM1_ESM.docx]

| Additional file 1A: Description of experimental assays carried out to study viral/viral coinfections and superinfections involved in PRDC (in vitro + ex vivo) | | | | | | |
| --- | --- | --- | --- | --- | --- | --- |
| **Coinfections** | **Protocols** | **Selected cells or tissues** | **Identified target cells** | **Viral interference** | **Immune responses** | **Reference PMIDs** |
| **swIAV**  **PRRSV** | swIAV: H1N1 Sw/Saskatchewan/18789/02  PRRSV: ISU-12-SAH  Interv: 3h/Inf: 15h +18h | NPTr-CD163 | NPTr-CD163 | - PRRSV reduced the replication of swIAV in coinfection and when inoculated 3h after. - swIAV decreased replication of PRRSV when inoculated 3h before. | - Both RNA viruses interfere with each other. - PRRSV primary infection has less effect than swIAV primary infection. - No clear synergistic nor additive effects observed - IFNλ1 are more expressed than other IFN. | 28757015 |
|  | swIAV: H1N1 Sw/Saskatchewan/18789/02  PRRSV: ISU-12-SAH  Interv: 3h/Inf: 18h | Lung slices  AM | swIAV: Epithelial cells  PRRSV: Pneumocytes type 1/MΦ  No coinfected cells | - PRRSV replication suppressed by swIAV - PRRSV did not alter swIAV replication in PCLS but reduced its replication in AMs. | - PRRSV reduced the response to swIAV. - swIAV showed low impact on PRRSV infection. - swIAV alters the ISGs expression but not PRRSV. - PCLS, synergy for TLR3, RIGl, and IFNβ expressions | 24418046 |
| **swIAV**  **PRCoV** | swIAV: H1N1 A/sw/Bad Griesbach/IDT5604/2006 and H3N2 A/sw/Bissendorf/IDT1864/2003  PRCoV: Bel85  Interv: 0h/Inf: 72h | NPTr  Lung slices | NPTr more permissive to swIAV than to PRCoV | - Viral titers in coinfections were lower than in single-infections. | - swIAV single- and coinfections with PRCoV showed stronger effects on ciliary activity than PRCoV single-infections. | 28779714 |
| **PCV2**  **PRRSV** | PCV2: Local field strain  PRRSV: JS-1  Interv: 2h/Inf: 6h to 96h | AM | AM permissive to both viruses | - PRRSV and PCV2 replication enhanced | - The coinfection induced IκBα degradation and phosphorylation 🡪 induced NF-κB activation. | 27080155 |
|  | PRRSV: VR2385  PCV2b: NC-16845  Interv: 0d/Inf: 24h | MoDC | MoDC | - ND | - DCs develop a regulatory phenotype upon PCV2 + PRRSV coinfection. - IL10 production increased in coinfection. - CD86 decreased in coinfection. - PDL1 increased in co- and PCV2 single-infection. | 26446939 |
|  | PRRSV: VR2385  PCV2a: ISU-40895  Interv: 0h/Inf: 36h | DC | DC | - ND | - DC permissive to PCV2 + PRRSV - PRRSV coinfection increased Treg lymphocytes. - TGFβ is upregulated in coinfection condition. | 22633482 |
|  | PRRSV: tw91  PCV2: Local strain  Interv: 0h/Inf: 18, 36, 54, 72, 90, 108h | AM | AM permissive to both viruses | - PRRSV infection rate was reduced in the presence of PCV2. However, the infectivity of PCV2 was unaffected. | - Cell death and apoptosis were reduced in coinfection conditions compared to PRRSV single infected AMs. - The reduction of PRRSV infection by PCV2 is related to the increased production of IFNα. | 15936905 |
| **PCV2**  **CSFV** | PCV2: HZ0201  CSFV: HCLV  Interv: 0/Inf: 72h | PK15  ST  AM | - PK15 and ST permissive to both viruses - AM permissive to PCV2 and not to CSFV | - PCV2 not influenced by CSFV - PCV2 suppressed CSFV. | - No effect of coinfection on apoptosis | 26431319 |

swIAV: swine Influenza A Virus, PRRSV: Porcine Reproductive and Respiratory Syndrome Virus, PCV2: Porcine Circovirus type 2 (subspecies a and b when indicated), CSFV: Classical Swine Fever Virus, PRCoV: Porcine Respiratory Coronavirus, AM: Alveolar Macrophage, NPTr: Newborn Pig Trachea cell line, DC: Dendritic Cells, MoDC: Monocyte Derived Dendritic Cells, PK15: Pig Kidney cells 15, ST: Swine Testicle cells, Interv: Interval, Inf: Duration of the infection assay

Additional file 1B Description of experimental assays carried to study viral/viral coinfections and superinfections involved in PRDC (in vivo)

| **Coinfections** | **Protocols** | **Clinical signs** | **Macroscopic lesions** | **Microscopic lesions** | **Other observations** | **Reference PMIDs** |
| --- | --- | --- | --- | --- | --- | --- |
| **PRRSV swIAV** | PRRSV: 10PL01  swIAV: H1N1 A/swine/Thailand/CU-PL65/2010  PRRSV then swIAV  Interv: 6 days | No major differences in clinical signs were registered except a prolonged fever in coinfected pigs compared to single-infected ones. | Coinfected group showed higher lung lesion scores than PRRSV single-infected group. | Interstitial pneumonia was more severe following infection with PRRSV. Additionally, coinfected animals showed epithelial necrosis and mononuclear cells infiltration in the alveolar septum. | -H1N1 viral load was more important in coinfected group compared to single-infected. | (no PMID)  The Thai Journal of Veterinary Medicine, 49(1), 71-79 |
|  | PRRSV: VR2385  swIAV: H1N1 A/Swine/IA/40776/92  PRRSV: at 4 and 7 wks of age  swIAV: at 7 wks of age | -Fever and reduced weight gain in coinfected animals  -Coinfected animals had the worst clinical disease. | Coinfected pigs showed a high percentage of swIAV-induced pneumonia. | Coinfection induced important interstitial pneumonia. | -No increase in swIAV shedding  -Increased lymphocyte proliferation after PRRSV infection  -PRRSV infection reduced swIAV vaccine efficacy. | 19595522 |
|  | PRRSV: Lelystad  swIAV: H1N1 A/Sw/Belgium/1/83  PRRSV then swIAV  Interv: 3, 7, and 14 days | -Differences in the clinical signs between coinfections and single infections were negligible. However, weight gain tends to be less important in coinfected animals. | ND | ND | -The time interval between both infections can affect the clinical outcome. Multiple infections with an interval of 3 or 7 days resulted in an acute disease while an interval of 14 days between the viruses resulted in a subclinical infection.  -Differences between coinfected and single infected pigs were negligible. | 15129583 |
|  | PRRSV: Lelystad  swIAV: H3N2  PRRSV then swIAV  Interv: 1 wk | ND | Coinfected pigs showed larger bronchiolar and lung lymph nodes than single infected. | Coinfection induced more pronounced inflammation of the bronchiolar wall. | PRRSV infection had no effect on swIAV infection. | 9220621 |
|  | PRRSV: Lelystad  swIAV: H1N1 A/Sw/Belg/ l/83  PRRSV then swIAV  Interv: 3 days | More severe clinical signs in coinfected animals than others | ND | ND | swIAV excretion delayed by PRRSV infection (2 days) | 9054128 |
|  | PRRSV: PL15-33  swIAV: H1N1 A/Poland/Swine/14131/2014  Simultaneous infections | Most severe clinical signs in the coinfected pigs | ND | ND | -The mean PRRSV load in BALF was higher in PRRSV-infected pigs than in coinfected pigs.  -IL6 and IL10 transcripts were upregulated in their expression in coinfected pigs but not in PRRSV-infected animals. | 31934657 |
| **PRRSV ADV** | PRRSV: EDRD-1 strain  ADV: YS-81 strain  PRRSV then ADV interv: 14 days | More severe clinical signs in coinfected pigs | Macroscopic lesions like brain congestion, pneumonia and thymus atrophy were more important in coinfected pigs. | Coinfected pigs showed more pronounced microscopic lesions (encephalitis, pneumonitis, tonsillitis and lymphadenitis). | ADV replication is enhanced by PRRSV. | 16423576 |
|  | PRRSV: EDRD-1 strain  ADV: YS-81 strain  PRRSV then ADV interv: 14 days | Clinical signs were more severe in the coinfected group. | Conifected pigs showed higher macroscopic lesions such as thymic atrohy, brain congestion and diffused pneumonia. | Neuronal microscopic lesions were more important incoinfected pigs. | Shedding of both viruses was enhanced in coinfected group. | 15511536 |
|  | PRRSV: E4 strain  ADV: Yamagata-S81 strain PRRSV then ADV  Interv: 7 days | Prolonged febrile response in coinfected pigs | Coinfected pigs showed mottled or diffusedly tan and red lungs with failure to collapse. | More severe pneumonic lesions in coinfected group | PRRSV excretion was higher with coinfection conditions. | 12710495 |
|  | PRRSV: Lelystad virus ter huurne ADV vaccine: strain M141  ADV: wild-type NIA-3  PRRSV then ADV vaccine then ADV challenge interv: 2 weeks then 8 weeks simultaneously | PRRSV pre-infected pigs only showed fever following ADV vaccination. | ND | Lymphoproliferative response to ADV was delayed and reduced but not inhibited in co-infected pigs. | Inhibition of ADV viral shedding by vaccination not affected by PRRSV | 10973691 |
| **PRRSV PRCoV** | PRRSV: Lelystad  PRCoV: 91V44  PRRSV then PRCoV  Interv: 3 days | More severe clinical signs in coinfected animals than others | ND | ND | PRCoV excretion not affected by PRRSV | 9054128 |
|  | PRRSV: SD23983  PRCoV: ISU-1  PRRSV then PRCoV  Interv: 10 days | Clinical signs were more severe in coinfected animals. | ND | ND | Coinfected pigs showed:   - -Synergistic decrease in NK- cytotoxicity. - -Higher production of IL6, IL10 and TGF-β than other pigs. - -Increased myeloid cells and CD4+CD8+ lymphocytes. | 20883160 |
| **PRRSV TTsuV1** | TTSuV1 natural infection  + PRRSV vaccination  + PRRSV challenge  Interv: 4 wks | Prior infection with TTSuV1 increases PRRSV clinical signs. | Macroscopic lung lesions more severe in case of TTSuV1 natural infection | ND | TTSuV1 suppresses immunization by PRRSV vaccines and exacerbates PRRSV clinical signs. | 22327391 |
| **PCV2**  **swIAV** | PCV2: ADDLPP 10069  swIAV: H1N1  PCV2 then swIAV  Interv: 7 days | Higher clinical scores in coinfected animals for a longer time | ND | No difference between coinfected and single-infected groups | - -swIAV did not affect the replication of PCV2.   -No difference in the antibody titer to PCV2 in the presence of swIAV | 20158948 |
| **PRRSV PCV2** | PRRSV: HF6-7  PCV2: THF601-7  Simultaneous infections | More severe clinical signs in coinfected pigs | More severe lesions in coinfected pigs | More severe lesions in coinfected pigs | - -Positive synergistic effect on the CD14 mRNA expression in coinfection condition | 26022073 |
|  | PRRSV: KS62  PCV2b: isolate 06-06274  Simultaneous infections | Coinfected pigs were divided into worst and best clinical outcome groups. The best outcome group consisted of pigs without any clinical disease. | The worst clinical outcome coinfected pigs showed interstitial pneumonia, granulomatous nephritis, multifocal fibrous adhesions in the abdomen and between the lungs. | In the worst clinical outcome groups, coinfection induced an interstitial pneumonia and a lymphoid depletion. | -PRRSV/PCV2 coinfection increased the rate of *Bacillus cereus* in the serum.  -PRRSV and PCV2 replication was higher in the worst outcome group.  -The microbial diversity was lower in the worst clinical group. | 27139023 |
|  | PRRSV: MLV vaccine  PRRSV: KS62  PCV2b: isolate 06-06274  Vaccination then simultaneous infections | Coinfection clinical signs were first reduced but increased later. | ND | After 22 days, PCV2 infection induced depletion of lymphocytes. | PRRS vaccination decreased PRRSV viremia but increased PCV2 viremia. | 26446422 |
|  | 4 PCV2 vaccines  PCV2b: SNUVR000463  PRRSV: SNUVR090851  Simultaneous infections post-vaccination | Coinfected group showed tachypnea, abdominal breathing, and severe dyspnea. No clinical signs in all vaccinated groups | Vaccinated groups showed less pulmonary lesions than coinfected non vaccinated group. | ND | -Coinfection prolonged severe clinical respiratory signs similarly to field PRDC.  -The vaccination against PCV2 was efficient in PCV2/PRRSV coinfected pigs. | 24403524 |
|  | PRRSV: HBR  PCV2b: YJ  PRRSV then PCV2b and PCV2b then PRRSV  Simultaneously or 7 days of delay | PRRSV/PCV2 group showed higher clinical scores than PCV2/PRRSV group. | PRRSV/PCV2 group showed the most severe haemorrhages. Dead pigs showed swollen, brown kidneys, and pulmonary congestion. | PRRSV/PCV2 group showed the most severe histological lesions. | -PRRSV/PCV2 group showed the highest viral loads and to the lowest antibody titers. | 23971711 |
|  | PRRSV: VR2385 and NC16845b  PCV2: 40895 and NC16845  Simultaneous infections | Coinfection induced more fever. | More severe macroscopic lung lesions for coinfected groups than others. | Increased levels of lymphocytes in coinfected pigs | -More severe lung lesions in coinfected pigs than others  -Stronger anti-PRRSV IgG response in response to coinfection | 22406346 |
|  | PRRSV: VR2385  PCV2: 4089  Simultaneous infections | No differences between groups | Coinfection induced higher severe lung lesions than PRRSV single infection. | Microscopic lesions were more severe in PCV2/PRRSV than single infection group. | / | 20637549 |
|  | PRRSV: VR2385  PCV2a: 40895  PCV2b: NC16845  Simultaneous infections | Coinfected pigs showed mild respiratory signs | Some coinfected pigs showed lymphohistiocytic interstitial nephritis. | Mild lung lesions in coinfected pigs | -PRRSV prolonged the infection with PCV2 and increased its replication.  -No differences between the two PCV2 subtypes | 21641124 |
|  | PCV vaccines  PCV2: 40895  PRRSV: ISU12  Simultaneous challenges | No difference in clinical signs between coinfected group and PRRSV single infected | Coinfected group showed the highest lung lesions. Vaccination reduced theses lesions in coinfected pigs. | The highest scores of microscopic lesions in the coinfected group animals. | -PCV2 single-infected pigs showed higher levels of Anti-PCV2-IgM than coinfected group. | 18430525 |
|  | PRRSV: HB-2 (sh)/2002  PCV2: BJ-HB  Simultaneous infections | Clinical signs in the coinfection group were more pronounced. | Gross lesions were more sever in coinfection group. | ND | -Coinfection prolonged the disease, increased the replication of both viruses, and decreased the antibodies production and the number of WBCs. | 18164875 |
|  | PRRSV: Olot/91  PCV2: /  PRRSV then PCV2  7 days of delay | Coinfected pigs suffered from growth retardation and fever. | ND | Coinfection has aggravated the lymphocyte depletion and the histiocytic infiltration. | -PRRSV increased the replication and the propagation of PCV2 in the lymphoid tissue and in the blood. | 18164875 |
|  | PRRSV: NADC-20  PCV2: 35358  Simultaneous infections | Coinfection mortality rate of 100% on day 20 compared to 26% for PCV2 and 0% for PRRSV alone | Coinfected pigs showed more pronounced hepatic lesions and severe proliferative interstitial pneumonia. | Microscopic lesions in coinfected pigs were also present in PCV2 single-infected pigs. | -ND | 11572560 |
| **PCV2 ADV** | ADV: YN1 strain  PCV2: ND Simultaneous infections | Coinfection increased clinical signs severity and mortality rate. | Higher neurologic and respiratory macroscopic lesions in the coinfected group | Microscopic lesions were more severe in coinfected pigs. | PCV2 increased ADV shedding in lungs and brain. | 31585642 |
| **PCV2**  **PPV** | PCV2: ISU-40895  PPV: NADL-8  2 PPV vaccinations before coinfection  Simultaneous infections | Coinfected animals had more pronounced fever. | Some coinfected pigs showed icterus, bleeding gastric ulcers, and pneumonia. | Lymphoid depletion was more important in coinfection conditions. | -Vaccination for PPV enhanced the replication of PCV2 in coinfected pigs.  -Vaccination against PPV did not affect the severity of PCV2 clinical signs. | 15036529 |
| **HEV PRRSV** | HEV: Genotype 3  PRRSV: Finistère  Half of the animals inoculated / half infected by contacts  Simultaneous infections | ND | Coinfection caused dramatic increase in HEV lesions at slaughter time | ND | -PRRSV increased HEV shedding.  -Immune response delayed  -PRRSV increased HEV transmission and infection susceptibility.  -Coinfection leads to chronic HEV infection. | 26048774 |
| **PorPV**  **swIAV** | H1N1: H1N1 A/Swine/New Jersey/11/76  PorPV: PAC-3  PorPV then H1N1  Interv: 44 days | Coinfected pigs showed increased clinical signs. | No macroscopic lesions were registered. | All groups presented interstitial pneumonia hyperplasia of the bronchiolar lymphoid tissue. | -Both viruses infect the bronchiolar epithelium.  -PorPV enhances the spread of H1N1 in the respiratory tissues without affecting the shedding. | 26854342 |

swIAV: swine Influenza A Virus, PRRSV: Porcine Reproductive and Respiratory Syndrome Virus, PCV2: Porcine Circovirus type 2 (subspecies a and b when indicated), CSFV: Classical Swine Fever Virus, PRCoV: Porcine Respiratory Coronavirus, TTsuV1: Torque Teno sus Virus 1, HEV: Hepatitis E Virus, PPV: Porcine Parvovirus, PorPV: Porcine Rubulavirus, WBCs: White Blood Cells, PRDC: Porcine Respiratory Disease Complex

Additional file 1C: Description of experimental assays carried to study bacterial/viral coinfections and superinfections involved in PRDC (in vitro + ex vivo)

| **Bacterium** | **Virus** | **Conditions** | **Cell types** | **Target cells** | **Replication/multiplication** | **Immune responses** | **Reference PMIDs** |
| --- | --- | --- | --- | --- | --- | --- | --- |
| *Mycoplasma hyopneumoniae* | PRRSV | HP-PRRSV: NJGC  Mhp: XLW-1  PRRSV then Mhp Interv: 1h, Duration: 6h, 15h | AM | AM | ND | -Several pathways are induced in coinfected cells. | 25445346 |
|  |  | PRRSV: VR2385  Mhp: 91-3  Duration: 24 and 48h | AM | ND | Mhp decreased the replication of PRRSV in AM at 24h. | -Increased production of IL1β, IL8 and IL10 in coinfection conditions | 11356254 |
|  | PCV2 | PCV2: 2010AHCY  Mhp: WX, AH, NJ, TH, XLW-1, and 168  PCV2 then Mhp, reverse, and simultaneous infections Interval: 0h to 24h, Duration: 72h | PK15 | Both pathogens detected in the same cell | PCV2 replication was enhanced by subsequent inoculation with Mhp but not by prior or simultaneous co-inoculations. | -ND | 27033909 |
| *Actinobacillus pleuropneumoniae* | PRRSV | PRRSV: IAF-Klop  App: S4074 serotype 1  PRRSV then App Interv: 4h, Duration: 48h | MARC-145 SJPL AM | 3 cell types permissive to PRRSV | PRRSV infection of SJPL and AM blocked by a pre-infection with App | -Enhanced cytotoxicity with coinfection -Anti-PRRSV activity due to App metabolites  -IFNγ contributed to this antiviral activity. | 24878741 |
|  |  | PRRSV: IAF-Klop  App: MBHPP147 from S4074  PRRSV then App culture supernatant  App culture supernatant for 2 hours then PRRSV then App culture supernatant  Interv: 4h, Duration: 48h | MARC-145 SJPL AM | 3 cell types permissive to PRRSV | Pre-treatment of AM with App culture supernatant reduced PRRSV replication in AM and SJPL but not in MARC-145 cells. | -The treatment of AM with App culture supernatant before the PRRSV infection decreased mRNA expression of IFNα and IFNβ. | 29293082 |
|  | PCV2 | PCV2: CC1  App: L20  PCV2 then App or App then PCV2 or simultaneous  Interv: 0h or 2h, Duration: 2h and 4h | AM | AM are infected by PCV2 and App. | App invasion and adhesion to AM was enhanced by PCV2. | -Clearance of App was reduced in coinfection conditions.  -Reduction of TNFα, IL4, and IFNγ production in coinfection conditions | 31176418 |
| *Glaesserella parasuis* | swIAV | swIAV: H3N2 A/swine/Spain/SF32071/2007  Hp: SW114 (serovar 3) or Nagasaki (serovar 5) strains  swIAV then Hp or Hp alone Interv: 1h, Duration: 1 or 8h | BMDC | -BMDC internalises Hp and swIAV. -Hp and swIAV in the same phagolysosome | ND | -No clear impact of swIAV pre-infection | 23157617 |
|  | PRRSV | PRRSV: Lelystad- CAPM V-490  Hp: HP 132 - CAPM 6475  PRRSV then Hp Interv: 24h, Duration: 28h and 48h | AM | AM | ND | -Increased IL1β, IL8, and TNFα production in coinfection situation  -Coinfection decreased ROS production. | 26358898 |
|  |  | PRRSV: Lelystad - CAPM V-490  Hp: HP 132 - CAPM 6475  PRRSV then Hp Interv: 24h, Duration: 28h and 48h | AM MDM | MDMs are more sensitive to PRRSV than AM. | Replication of PRRSV not affected by Hp but coinfection reduced the growth of Hp. | -Coinfected macrophages produced less ROS than PRRSV single-infected cells. | 28472979 |
|  |  | PRRSV: Lelystad Hp: HP 132 - CAPM 6475 PRRSV then Hp  Interv: 24h, Duration: 28h and 48h | AM  MDM | MDMs and AMs are permissive to PRRSV. | MDMs mortality is not affected by Hp in coinfection conditions. | -Coinfection of MDMs:  Reduced gene expression and production of IL1β and IL8 compared to single infections  Reduced mRNA expression of CD86, CD14, and CXCL10 compared to Hp single infection  Increased mRNA levels of TNFα  -Unlike AMs, MDMs increased expression of IFNα following PRRSV infection. | 30322536 |
| *Streptococcus suis* | swIAV | swIAV: H1N1 A/swine/St-Hyacinthe/148/1990  Ss: 31533  swIAV then Ss  Interv: 12h, Duration: 12h | NPTr | ND | ND | -CCL5, IL8, VCAM1, and COX2 significantly more upregulated in the presence of both pathogens compared to single-infection | 24708855 |
|  |  | swIAV: H1N1 A/sw/Bad Griesbach/IDT5604/2006 and H3N2 A/sw/Herford/IDT5932/2007  Ss: 10  swIAV + Ss or Ss alone Interv: 0h, Duration: 72h | Lung slice | Ss selectively attached to swIAV-infected cells. | -swIAV promoted bacterial adherence and colonization. -Bacterial adherence facilitated by swIAV  -swIAV-impairment of the mucociliary barrier plays a major role in promotion of bacterial infection. | ND | 25916988 |
|  |  | swIAV: H3N2 A/swine/St-Hyacinthe/148/1990  Ss: 31533 swIAV then Ss  Interv: 12h, Duration: 24h | NPTr | Colocalization of Ss and swIAV | Bacterial adhesion and invasion significantly increased by swIAV | -Higher levels of CCL2, CCL4, IL6, IL8, and TNFα in coinfected cells than in mon-infected | 24082069 |
|  |  | swIAV: H1N1 A/sw/Potsdam/15/1981, H1N1 A/sw/Bad Griesbach/IDT5604/2006, H3N2 A/sw/Herford/IDT5932/2007, H3N2 A/sw/Damme/IDT5673/2006, H1N1 A/Swine/Shanghai/1/2005  Ss: 10 and 10 cps∆EF  swIAV then Ss  Interval: 2h or assay: up to 72h | NPTr | Co-localization of Ss and swIAV | -swIAV enhanced bacterial adhesion.  -Ss adhesion increased via the capsular α-2,6-linked sialic acid recognized by HA expressed by swIAV-infected cells -Bacterial coinfection had a negative effect on the replication of swIAV. | ND | 26297001 |
|  | PRRSV | PRRSV: IAF-Klop  Ss: P1/7  PRRSV then Ss Interv: 2h, Duration: 5h, 12h | BMDC Monocytes | -BMDC more permissive to PRRSV than monocytes -Ss intracellular | -PRRSV impaired the phagocytosis of Ss. | - Increased pro-inflammatory response of BMDC to Ss after a pre-infection with PRRSV  -Additive effects for CCL4, CCL14, CCL20, and IL15 -Synergistic effects for IL6 , CCL5, and TNFα -Little effect of PRRSV pre-infection on monocyte response to Ss | 27213692 |

BMDC: Bone Marrow Dendritic Cell, NPTr: Newborn Pig Trachea, PK15: Porcine Kidney 15, MDMs: Monocyte Derived Macrophages, ROS: Reactive Oxygen Species, SJPL: St-Jude Porcine Lung cell, swIAV: swine Influenza A Virus, PRRSV: Porcine Reproductive and Respiratory Syndrome Virus, PCV2: Porcine Circovirus type 2 (subspecies a and b when indicated)

Additional file 1D: Description of experimental assays carried to study bacterial/viral coinfections and superinfections involved in PRDC (in vivo)

| **Bacterium** | **Virus** | **Protocols** | **Clinical signs** | **Macroscopic lesions** | **Microscopic lesions** | **Pathogen detection** | **Results** | **Reference PMIDs** |
| --- | --- | --- | --- | --- | --- | --- | --- | --- |
| ***Mycoplasma hyopneumoniae*** | **swIAV** | swIAV: H1N1 A/Swine/IA/40776/92  Mhp: 232  Mhp then swIAV Interv: 21 days | Coinfected pigs coughed significantly more than the others. | More severe pneumonia in coinfected pigs than in others | No difference | Similar distribution | No impact on antibody levels in serum | 11427564 |
|  |  | swIAV: H1N1 A/Sw/Hok/2/81  Mhp: E-1 Mhp then swIAV Interv: 7/21 days | Clinical signs (coughing and fever) were more observed in coinfected pigs. | H1N1 infection, 7 or 21 days after Mhp infection, resulted in a greater percentage of dark red-lung lesions. | Bronchial epithelial lesions and interlobular oedema only observed in the dual infection groups | -No impact of swIAV on the Mhp titers  -No impact of Mhp on virus duration shedding | No impact of Mhp on the antibody levels against H1N1 | 15036530 |
|  |  | swIAV: H1N1 A/Sw/Cotes d’Armor/0231/06 and H1N2 A/Sw/Cotes d’Armor/0113/06  Mhp: 116  Mhp then swIAV Interv: 21 days | Mhp pre-infection increased influenza clinical signs. | Mhp increases the mean lesion score for the Mhp+H1N1 group not for the Mhp+H1N2 group. | Exacerbation of bronchial pneumonia and superinfection with cellular exudates in the alveoli, more marked in the lungs of Mhp+H1N1 group than in Mhp+H1N2 group | -No impact of Mhp on both swIAV shedding nor on H1N2 multiplication in lungs  -At 7dpi H1N1 detected in co- but not in single inoculated pigs  -Less Mhp in the Mhp+H1N2 group than in the Mhp or Mhp+H1N1 groups | Higher humoral immune response to H1N2 infection in the Mhp+H1N2 group than in the H1N2 group at 7dpi swIAV | 22261237 |
|  |  | swIAV: H1N1 A/Sw/Cotes d’Armor/0231/06  Mhp: 116  Mhp then swIAV Interv: 21 days No single-infected group in this study | Some clinical signs were observed in the coinfected group (fever, cough and decreased mean weight gain). | Extensive pneumonia lesions | bronchiolitis, broncho-interstitial, and interstitial pneumonia | Detection of both pathogen in all lung lobes | Mhp infection induced an oxidative stress before H1N1 infection. | 23266108 |
|  |  | swIAV: H1N1 A/Sw/Cotes d'Armor/0231/06  Mhp: 116  Mhp then swIAV Interv: 21 days No single-infected group in this study | Feed-restricted pigs presented shorter hyperthermia and a positive mean weight gain over post-H1N1 infection whereas animals fed *ad libitum* lost weight. | All coinfected pigs had macroscopic lesions of pneumonia. | All coinfected animals developed bronchiolitis, broncho-interstitial and interstitial pneumonia. | -Mhp genome detected in all lung lobes -swIAV genome detected in nasal swabs and lung tissues | Both infection and feed restriction reduced postprandial glucose concentrations, indicating changes in glucose metabolism. | 25101681 |
|  |  | swIAV: H1N1 A/Sw/Cotes d’Armor/0231/06  Mhp: 116  Mhp then swIAV Interv: 21 days No Mhp single-infected group in this study | No impact of the Mhp pre-infection on clinical signs usually induced by swIAV | No impact of the Mhp pre-infection on pneumonia lesions | Earlier and more extended inflammatory lesions in coinfected compared to single-infected pigs | No impact of the Mhp pre-infection on viral excretion and multiplication | Coinfected pigs presented an earlier influx of CD163+ cells in cardiac lobes and a greater influx of neutrophils and of pro-inflammatory cytokines in BALF than single-infected pigs. | 27498789 |
|  | **PRRSV** | PRRSV: MN-30100 and MN-184  Mhp: 232  Mhp then PRRSV  Interv: 21 days | Coinfection exacerbated the disease and increases the duration and the severity of the clinical signs. | ND | ND | Mhp increased the replication of PRRSV in lymphoid tissue and blood. | ND | 16506914 |
|  |  | PRRSV: VR2385  Mhp: 11 Interval: few hours | Coinfection PRRSV/Mhp increased clinical signs of respiratory disease. | Dual infection PRRSV/Mhp increased pneumonia lesions. | Microscopic lesions (Mhp) were most severe in coinfected pigs. | No observation regarding coinfections | No observation regarding coinfections | 10649626 |
|  |  | PRRSV: VR2385  Mhp: 232  Interv: 0 | Clinical disease was more severe in coinfected pigs. | Pneumonia was more frequent and pronounced in coinfected pigs. | No difference registered between single and dual infections | -PRRSV detection was prolonged in the presence of Mhp.  -Mhp infection was not altered by PRRSV. | -IL12 and IL10 transcript expressions were higher in coinfected pigs than in single-infected.  -Higher production of IFNγ and IL10 in BAL fluids in coinfected animals than others | 14583150 |
|  |  | PRRSV: VR2385  Mhp: 11  Mhp and PRRSV simultaneously or with an interval: -Mhp 21 days before PRRSV -Mhp 10 days after PRRSV | Pigs infected with both Mhp and PRRSV had more severe clinical. respiratory disease. | Coinfection induced an acute interstitial pneumonia. | PRRSV infection increased the microscopic lesions typical of Mhp. | -No evidence of increased numbers of cells containing PRRSV antigen in tissues with Mhp lesions  -No significant differences in Mhp titer between groups | -PRRSV infection increased the severity of the Mhp-induced pneumonia.  -Mhp-infected pigs showed increased PRRSV-induced pneumonia lesions. | 9986823 |
|  |  | PRRSV: VR2385  Mhp: 232  Simultaneous infections | Increased clinical respiratory disease and slower viral clearance | Coinfection increases the percentage of lung showing visible lesions. | ND | ND | -The pigs coinfected with PRRSV and Mhp had increased levels of IL1β, IL8, IL10, IL12, and TNFα. | 15358650 |
|  |  | PRRSV: MN-30100  and MN-184 Mhp: 232  Simultaneous infections | Coinfection with PRRSV and Mhp increased the severity of the clinical disease. | ND | ND | -Large quantities of virus were detected in the aerosols | -Coinfection did not influence the concentration of PRRSV in aerosols. | 17042383 |
|  |  | PRRSV: IND-5  Mhp: P5722-3  Interv: 7 days | No difference between single and coinfected pigs | ND | No difference between single and coinfected pigs | ND | -PRRSV infection did not increase the severity of Mhp infection in piglets. | 8734647 |
|  | **PCV2** | PCV2: ISU-40895  Mhp: 232  Mhp then PCV2  Interval: 14 days | Dual-infected pigs had moderate dyspnea, lethargy, and reduced weight gain. | Increased severity of pneumonia lesions in coinfected pigs | Higher PCV2-associated microscopic lesions in lung and lymphoid tissues of coinfected pigs than others | -Mhp pre-infection increased the amount of PCV2. | -Higher serum antibody response to Mhp and higher antibody response to PCV2 in dual-infected pigs | 15557072 |
|  |  | PCV2: ISU-40895  Mhp: 232  Mhp then PCV2 Interv: 14 days | Dual-infected pigs showed mild-to-severe respiratory disease. No difference with Mhp single-infected pigs | Pigs infected with Mhp (alone or in association) had higher lung lesion scores than pigs infected with PCV2. | Lymphoid depletion and histiocytic infiltration in tracheobronchial lymph nodes in groups infected with PCV2 | -No impact of Mhp on PCV2 detection in sera | -No effect of coinfections | 21176971 |
|  |  | PCV2: I-12/11  Mhp: 98 Simultaneous infections | No impact of infections | No differences in the mean lung score detected between Mhp-inoculated groups | Mild microscopical lesions in one or two pigs infected with PCV2 (single and coinfections) | -No differences in PCV2 load or duration of viremia between groups -No differences in Mhp load in nasal swabs between groups | -No impact of Mhp on PCV2-antibody titer -No impact of PCV2 on Mhp seropositive pigs | 22522076 |
|  |  | PCV2: SNUVR000463  Mhp: SNU98703 Interv: 14 days | ND | Higher gross lung lesions in the dually infected groups | Dually infected pigs had more severe PCV2-induced pulmonary and lymphoid lesions than others.  Coinfection did not significantly potentiate the Mhp-induced pulmonary lesions. | -Dually challenged pigs had increased PCV2-viremia but no difference in Mhp nasal shedding. | -No impact of the coinfection on the immunological responses against Mhp  -Higher immunological responses against PCV2 in the PCV2 single-infected group compared to the coinfected group | 24631087 |
| ***Actinobacillus pleuropneumoniae*** | **swIAV** | swIAV: H1N1 A/Poland/Swine/14131/20141  App: 4226 serotype 2  Simultaneous infections | Clinical signs were more severe in the coinfected group. | Macroscopic lesions were more severe in the coinfected group. | ND | ND | Increased concentrations of IL1β, IL8, and IFNα in the lungs of coinfected animals | 29978082 |
|  |  | swIAV: H1N1 A/Poland/Swine/14131/2014  App: PIWetHps192/2015  Simultaneous infections | Clinical signs were more severe in the coinfected group. | swIAV-like lesions were more severe in coinfected pigs. | ND | -Coinfection enhanced the nasal swIAV shedding and virus replication. -No impact of swIAV on App load and shedding | -Earlier detection of anti-HI antibodies in the coinfected group -Strongest SAA and Pig-MAP responses in coinfected pigs | 29202835 |
|  | **PRRSV** | PRRSV: LV-Ter Huurne  App: 17415  Interv: 8 days PRRSV then App | ND | ND | ND | ND | -The infection affected the IgM, not the IgG isotype. | 29126442 |
|  |  | PRRSV: Lelystad  swIAV: H3N2 App: 1421  -PRRSV/swIAV: Interv: 7 days PRRSV then swIAV -PRRSV/App: Interv: 2 days App then PRRSV | PRRSV/H3N2: PRRSV infection does not aggravate the acute stage of H3N2 infection but could make influenza infection more chronic.  -Coinfection PRRSV/App:  App produced more severe disease. | Coinfection PRRSV/H3N2: Inflammation of the bronchiolar wall more pronounced | ND | -PRRSV-positive macrophages in the lungs and rare influenza-positive cells in the bronchiolar epithelium  -App lesions were surrounded by PRRSV-positive macrophages. | -No impact of a previous PRRSV infection on the acute of chronic stage of influenza infection  -Lung alveolar macrophages and bronchiolar epithelial cells do not become more susceptible for infection after App infection. | 9220621 |
|  |  | PRRSV: LV-Ter Huurne App: 17415  PRRSV then App  Interv: 8 days | ND | Coinfection significantly increased the patho-histological score. | Coinfection increased lympho-monocytic cell infiltration in the lung. | ND | ND | 27606818 |
|  | **ADV** | ADV: strain YS-81  App: ZF-867 serotype 1 ADV then App  Interv: 7 days | Coinfected pigs showed more severe clinical signs, especially fever. | Pneumonic lesions were more important in coinfected pigs. | ND | ADV shedding was increased in presence of App. | App increased the severity of ADV infection in pigs. | 8399736 |
| ***Bordetella bronchiseptica*** | **swIAV** | swIAV: H1N1 A/Swine/Minnesota/37 866/1999 (MN99)  Bb: KM22  Simultaneous infections | ND | Pneumonia lesions were more severe in the coinfected group. | Lesions were more severe in the coinfected group (peribronchiolar lymphocyte infiltration, accumulation of neutrophils, and alveolar epithelial cell necrosis) | -No impact of coinfection on swIAV shedding but enhancement of Bb burden in the coinfected group. | -Higher type I IFN response and enhanced IL1β and IL8 mRNA expression in coinfected group than others | 20558274 |
|  |  | swIAV: H1N1 A/Swine/Minnesota/37 866/1999  Bb: KM22  Simultaneous infections | ND | ND | ND | ND | Coinfection induced an enhanced expression of TLR3. | 21561668 |
|  |  | swIAV: H1N1 A/sw/Poland/KPR9/2004  Bb: field strain  Simultaneous infections | Hyperthermia for a longer period in the coinfected group than others | More severe and extensive lesions in coinfected piglets than others | ND | -No impact of swIAV on Bb in lung  -Bb infection delays the swIAV clearance in the lung. | -Higher mRNA levels for IFNα and IL8 in BALF of coinfected pigs than others | 24629899 |
|  |  | swIAV: H1N2 A/swine/Minnesota/03012/2010  Bb: KM22  Interv: 28 days | ND | Higher percentage of the lung tissue affected in the coinfected group than others | More severe in coinfected pigs than others | -No impact of swIAV on the Bb respiratory tract colonization  -No impact of Bb on virus multiplication in the lung | -Higher level of MCP1 in the coinfected group compared to single-infected groups | 30337924 |
| ***Pasteurella multocida*** | **swIAV** | swIAV: H1N1 A/sw/Poland/KPR9/2004  Pm: field strain  Simultaneous infections | Coinfected pigs had clinical signs (no single-infected). | -Lung lesions observed at 3, 5, and 10 days post-infection  -Atrophy of turbinates observed at 10 days post-infection only | ND | -Viral shedding from 2 to 7 dpi  -No virus detected at 10 dpi  -Pm detected in nasal swabs from 3 to 10 dpi and lungs | -Increased concentrations of CRP, SAA, haptoglobin, and MAP in serum | 23332090 |
|  |  | swIAV: H3N2 Sw/Ghent/172/2008  Pm: field strain  Simultaneous infections | -All coinfected pigs had clinical signs.  -Less severe disease than after H1N1+ Pm coinfection [73] | Various lesions in the coinfected pigs | ND | -Virus detected in nasal swabs and lungs in the first days after inoculation  -Pm detected in nasal swabs and lungs | -Lower levels of CRP, SAA, and MAP than after H1N1+Pm coinfection [73] | 26161700 |
|  | **PRRSV** | PRRSV: NADC-21  Pm: P-3480  Bd: KM22  PRRSV then Pm  Interv: 7 days | Coinfected animals showed no difference in clinical signs from single-infected ones. | No difference between coinfected and single-infected regarding gross lesions | ND | No effect of coinfection on pathogens’ replication | ND | 11327458 |
|  |  | PRRSV: VR2332  ADV: 4892  Pm: A52 and A24  Interv: 5 days  PRRSV then Pm  ADV then Pm | Central nervous clinical signs detected only in animals challenged with ADV | ADV/Pm: More extensive pneumonic lesions than in other groups | -ADV/Pm and PRRSV/Pm: interstitial pneumonia, catarrhal purulent bronchopneumonia and polymorphonuclear neutrophils in alveoli -ADV/Pm: Meningo-encephalitis and purulent rhinitis | ND | -PRRSV/Pm, pneumonic lesions very slight compared to ADV/Pm  -The role of PRRSV on the development of pulmonary lesions is unclear. | 9220619 |
| ***Glaesserella parasuis*** | **swIAV** | swIAV: H1N1 A/Poland/Swine/14131/2014  Hp: PIWetHps192/2015  Simultaneous infections | No differences between swIAV and swIAV/Hp groups | No differences between swIAV and swIAV/Hp groups | ND | -No effect of swIAV on the Hp shedding but Hp infection increased swIAV shedding.  -Enhanced Hps and swIAV lung replication in coinfected animals | -More important increase in cytokine production (IFNγ, TNFα, IL1β, IL6 and IL10) and APP (haptoglobin, C-reactive protein, SAA and MAP) after coinfection | 29202835 |
|  | **PRRSV** | PRRSV: VR2332, SDSU73, rJXwn06, rSRV07  Cocktail of Hp, *Streptococcus suis*, and *Actinobacillus suis* Bacteria then PRRSV, 1 week later | -More severe clinical signs in coinfected groups  -Severity was also dependent of the PRRSV strain. | ND | Bacteria/PRRSV: bronchopneumonia with alveoli and larger airways containing neutrophils and/alveolar necrosis | ND | -PRRSV infection causes interstitial pneumonia and predisposes to secondary bacterial bronchopneumonia in a strain dependent manner. -The severity of disease could depend of the virulence of the PRRSV strain and the development of secondary bacterial infection. | 28619168 |
|  |  | PRRSV: HuN4 HUN4  Hp: Nagasaki  Only alveolar macrophages were coinfected | / | / | / | -Bacterial sequencing in broncho-alveolar lavage: 11 bacterial species were increased after PRRSV infection, including Hp. | Hp proliferates faster in PRRSV-infected piglets. | 28532803 |
|  |  | PRRSV: SX-1  Hp: LZ  Interv: 5 days  PRRSV then Hp | -Single- and coinfections increase rectal temperatures.  -Higher temperatures in coinfected pigs than others  -High mortality in coinfected pigs | More lesions of the ears and dorsum in coinfected pigs | Coinfection PRRSV/Hp leads to severe interstitial pneumonia. | -Higher Hp loads were observed in coinfected pigs than in single-infected pigs. | -Coinfection increases the amount of Hp in blood at 3 days post-infection.  -PRRSV could accelerate HP infection. | 22460022 |
|  | **PCV2** | PCV2: WH (2b)  Hp: MD0322  Simultaneous infections | Coinfected piglets developed dyspnea, anorexia, prostration, and weight lost (more than single-infected pigs) | ND | Coinfection resulted in severe widened alveolar septa, lymphocyte infiltrations in lungs, and lymphocyte depletion. | -Increased amount of virus in serum and tissues in coinfected pigs | -Coinfection resulted in slower generation and lower levels of anti-PCV2 antibodies.  -Lower numbers of blood leukocytes in coinfected pigs than others | 29157279 |
| ***Streptococcus suis*** | **swIAV** | swIAV: H1N1 A/swine/Hubei/101/2009  Ss: 05ZY  Interv: 3 days  swIAV then Ss | More severe clinical signs in the coinfected group than in other groups | More serious lung lesions in the coinfected groups than in other groups | More important lung damages (extensive cellular infiltrates, bleeding, and cellular debris) in the coinfected pigs than others | -Higher viral titers in nasal swabs and lung 6 days post-infection in the coinfected pigs than others  -No impact of swIAV on the bacterial loads | Genes related with the immune response and apoptosis were highly overexpressed in the coinfected group. | 25906258 |
|  | **PRRSV** | PRRSV: VR2385  Ss: ISU VDL 40634/94 -  Interv: 7 days  PRRSV then Ss | Highest mortality rate in the dually infected group | No significant differences between single- and coinfected groups | No significant differences between single- and coinfected groups | Presence of PRRSV and Ss more important in dually infected pigs | ND | 10714643 |
|  |  | PRRSV: HuN4  Ss: WC0711 Simultaneous infections | Coinfected pigs died of an acute disease. | Several pigs in the coinfected group had pleuritis. | Severe lesions in died pigs | -Bacteremia and viremia were observed in all the coinfected pigs. | -The coinfection increased the pathogenicity and the mortality. | 20696031 |
| ***Mycoplasma hyorhinis*** | **PRRSV** | PRRSV: LMY  Mhr: EH5  Simultaneous infections | / | The lung tissue from coinfected animals showed more severe histopathological signs. | The lung tissue from coinfected animals showed more severe histopathological signs. | ND | -More severe histopathological signs in coinfected piglets than in others | 27436444 |
|  | **PCV2** | PCV2: YJ  Mhr: DL  PCV2 then Mhr or Mhr then PCV2 or simultaneous infections Interv: 0 to 7 days | Lower average daily weight gains in coinfected pigs than in others | Dual infection resulted in more severe macroscopic lung lesions. | Dual infection resulted in more severe microscopic lung lesions. | -Larger amounts and wider range of tissue distribution of PCV2 in coinfected pigs than in others | -Higher levels of PCV2 and Mhr antibodies in the coinfected groups -Significant increase of TNFα, IL2, and IL6 in coinfected pigs compared to single-infected | 26711038 |
| ***Staphylococcus aureus*** | **swIAV** | swIAV: H3N2 A/Swine/Minnesota/1145/2007  Sa: NRS123  swIAV then Sa Interv: 3 to 6 days | Animals infected with Sa 5 days after swIAV infection showed a second increased body temperature. | Dually infected animals showed more red hepatization of the lung than other animals. | No difference between H3N2 and H3N2/Sa groups | -Only dually infected animals had viable Sa in the lung, lymph nodes, and spleen 48 h after bacterial infection. | Sa impacted swIAV infection. | 23074662 |

Interv: Interval, dpi: days post-infection, ND: Not Determined, ADV: Aujeszky’s Disease Virus, swIAV: swine Influenza A Virus, PRRSV: Porcine Reproductive and Respiratory Syndrome Virus, PCV2: Porcine Circovirus type 2 (subspecies a and b when indicated)

Additional file 1E: Description of in vivo experimental assays carried to study the bacterial/bacterial infections and superinfections involved in lung diseases of PRDC

| **Bacterial species** | **Protocol / interval between infections**  **(CI or SI*)** | **Clinical signs** | **Macroscopic lesions** | **Main laboratory results** | | | **Reference PMID** |
| --- | --- | --- | --- | --- | --- | --- | --- |
|  |  |  |  | **Microscopic lung lesions** | **Bacterial detection** | **Immune responses** |  |
| *M. hyopneumoniae* (Mhp) and  *A. pleuropneumoniae* (App) | -Mhp and App serotype 9 / 0 day (CI)  -App serotype 9 then Mhp / 28 days (SI)  -Mhp and App serotype 9 / 28 days (SI)  -Duration of the experiment: 30-65 days | In the three groups: mortality, hyperthermia, severe coughing, and decreased average daily weight gain.  *App* infection is potentiated by *Mhp*. | In the three groups: pneumonia, pleurisy, fibrinous and haemorrhagic pleuro-pneumonia, pulmonary necrosis, and hypertrophy of the tracheo-bronchial lymph nodes | In the three groups: lesions of early phase of App infection, haemorrhage, vascular thrombosis, oedema, necrosis and the presence of fibrinous exudate / lesions of chronic phase of App infection, marked fibrosis around areas of necrosis and fibrinous pleuritis / lesions of Mhp infection, infiltrating lymphocytes in the peribronchiolar area, interstitial pneumonia, lymphoid nodules, and collapse of the alveoli | *Mhp* and *App* were detected by PCR and culture without further information on multiplication. | -In the three groups: IgG anti-Mhp and anti App serotype 9  -2–3 weeks after infection,  similar ELISA titers | 18977616 |
|  | -Mhp then App serotype 5/ 14 days (SI)  -Mhp then App serotype 5/ 28 days (SI)  -Mhp then App serotype 5/ 35 days (SI)  -Duration of the experiment: 6 weeks - + 3 days | ND | ND | ND | ND | Phagocytosis suppression is more important in dually infected pigs. | 2301832 |
|  | -Mhp then App type I / 7 days (SI)  -Duration of the experiment: 14 days | Higher mortality and clinical signs in App single infected animals | Fibrinous and haemorraghic pleuro-pneumonia are less severe in pigs pre-infected with Mhp compared to App single infected pigs. | ND | ND | -IgG anti App: seropositivity 1 week after infection  -Titers of these IgG were lower in double infected pigs compared to App single infections. | 2531628 |
|  | -Mhp then App /16 days (SI)  -Duration of the experiment: 35 days | Mortality and severe clinical signs in coinfection conditions | Coinfections showed more extensive and widespread pneumonic lesions than single-infections. | Haemorrhagic fibrinous pleuropneumonia,  chronic form with necrotic nodules | -Mhp and App titers in the upper respiratory tract were higher in dually infected animals | No correlation between antibody titers to App and intensity of the lung lesions | 6513243 |
| *M. hyopneumoniae* and *P. multocida* (Pm) | -Mhp then Pm / 23 days (SI) | -Aggravated fever, cough and dyspnea in dually infected animals  -Similar ADWG but dually infected pigs consumed 60% more food. | Extensive exudative pneumonia | Enlarged septa, large increase in the number of PMN in alveoli and bronchi, alveolar and interstitial haemorrhages, marked alveolar macrophage proliferation, and perivascular and alveolar lymphocytic infiltration with fibrin deposition | -Pm isolated exclusively from dually infected pigs  -Mhp positive pigs were higher in dually infected animals. | ND | 3196973 |
|  | -Mhp then Pm / 13 weeks (SI) – group 1  -Mhp vaccination (3 and 8 week-old) then Mhp inoculation then Pm / 4 weeks respectively (SI) – group 2  -Mhp inoculation then Pm / 4 weeks (SI) – group 3  -Pm alone – group 4  -Duration of the experiment: 15 weeks | -Coughing in all groups with the highest frequency of coughing in Mhp and Pm inoculated group (group 3)  -Dyspnea in group 3 (Mhp+Pm) | Pneumonia in all groups with the most severe lesions in dually infected group 3 | -Peribronchiolar lymphocytic inflammation in all groups (lowest score in group-4 pigs)  -Alveolitis in all groups (highest score in group-3 pigs) | Mhp induced the multiplication of Pm since Pm was detected in dually infected pigs of group 3 only but not in single infected or dually infected vaccinated pigs against Mhp of group 2.  -Percentage of lung surface area with pneumonia correlated with the number of Pm colonies isolated in group 3. | Seroconversion to Mhp in all groups with varying frequencies of seropositive pigs | 8125807 |
|  | -Mhp then Pm / 20 days (SI) | Coughing | Pneumonia | Bronchial and perivascular lymphoid accumulations in some pigs | Mhp  Pm  App in nasal swabs | Seroconversion to App was higher in pigs pre-infected with Mhp. | 22632286 |
|  | -Mhp then Pm / 13 days (SI) | Coughing without significant differences between groups | Pneumonia in all the groups | ND | Mhp multiplication is not affected by App. | ND | 28499212 |
| *M. hyopneumoniae* and *P. multocida* and *A. pleuropneumoniae* | -Mhp then Pm then App serotype 2 / 7 days and 7 days, respectively (SI) | Sneezing, depression, forced breathing, coughing, and elevated rectal temperature at some points after Pm or App infections | Catarrhal pneumonia and purulent foci in the diaphragmatic lobes | -Lymphohistiocytic bronchitis and  peribronchitis and interstitial pneumonia  peribronchitis  -Hyperplasia of the peribronchial lymph nodes, lymphohistiocytic, and infiltration of the nasal mucosa | Mhp and App detection | Seroconversion to Mhp | 11768127 |
| *M. hyopneumoniae* and *M. hyorhinis* (Mhr) | -Mhp at 6 weeks of age then Mhr at 7 weeks of age / 7 days (SI)  -Duration of the experiment: 33-36 days | Coughing,  Hyperthermia, and  lower ADWG during the third week | Pneumonia, pleuritic, and pericarditis | ND | Mhp and Mhr (PCR) | -IgG anti-Mhp: seropositivity from 13 dpi  -Higher haptoglobin concentration  (additive effect) | 31030844 |
| *M. hyopneumoniae* and *M. flocculare* (Mfloc) | -Mhp at 6 weeks of age then Mfloc at 7 weeks of age / 7 days (SI)  -Duration of the experiment: 33-36 days | Coughing and  lower ADWG during the third week | Pneumonia | ND | Mhp and Mfloc (PCR) | -IgG anti-Mhp: seropositivity from 13 dpi -Higher haptoglobin concentration (additive effect) | 31030844 |
|  | -Mhp and Mfloc / 0 days (CI)  -Mfloc then Mhp / 6 weeks (SI)  -Duration of the experiment: 7 to 24 weeks post-infection | Clinical signs following Mhp infection such as coughing are unchanged in presence of Mflo | No difference registered between dually infected and Mhp single infected pigs | Histopathology revealed characteristic pneumonia induced by Mhp in all pigs without any difference between single and dually infected groups. | -Mhp and Mfloc were detected by IFA and cultutre.  -The poor colonization of the respiratory tract by Mfloc was not affected by Mhp. | Mhp induced a stronger antibody response that was not influenced in the presence of Mfloc in dually infected groups. | 1570675 |
| *B. bronchiseptica* (Bb) and *H. parasuis* (Hps) | Bb and Hps / 0 day (CI)  Bb then Hps / 7 days (SI)  Duration of the experiment: 2 weeks | Coughing was reported in case of Bb and/or Hp infection while Bb caused sneezing in infected pigs. | -Pneumonia: areas of tan-red consolidation with well-demarcated borders and a cranial ventral distribution (no statistical differences between the groups) | ND | Bb predisposes to Hps colonization while Hps reduced the multiplication of Bb in the nasal cavity. | ND | 15019114 |
| *B. bronchiseptica* and *P. multocida* | Bb then Pm / 4 days (SI)  Duration of the experiment: 25 days | ND | Atrophic rhinitis scores were higher in dually infected compared to Pm single infected pigs. | ND | Bb enhanced the multiplication of Pm. | ND | 17624695 |
|  | Bb then Pm / 7 days (SI)  Duration of the experiment: 25 days | Clinical signs such as sneezing, coughing, fever and weight loss were higher in dually infected group compared to Pm single infected group. | Atrophic turbinates and mucopurulent nasal discharge were exclusively noted in animals pre-infected with Bb. | ND | Bb enhanced the multiplication of Pm in the upper respiratory tract. | ND | 11327458 |

ADWG: Average Daily Weight Gain, CI: Coinfection, IFA: Immunofluorescence assay, SI: Superinfection, swIAV: swine Influenza A Virus, PRRSV: Porcine Reproductive and Respiratory Syndrome Virus, PCV2: Porcine Circovirus type 2 (subspecies a and b when indicated), PRDC: Porcine Respiratory Disease Complex
